# Combining polydioxanone-based scaffold and allogeneic adipose-derived mesenchymal stem cells enhances articular cartilage repair - a pre-clinical study

**DOI:** 10.1016/j.jot.2026.101193

**Published:** 2026-08-25

**Authors:** Emanuel Vitor Appolonio, Vittoria Guerra Altheman, Fernanda de Castro Stievanni, Gustavo dos Santos Rosa, Fernanda Barthelson Carvalho de Moura, Natalia Camargo Faraldo, Carlos Eduardo Fonseca Alves, Camila C. Kaleka, Pedro Debieux, Moises Cohen, Ana Liz Garcia Alves

**Affiliations:** a Department of Veterinary Surgery and Animal Reproduction, Regenerative Medicine Lab, School of Veterinary Medicine and Animal Science, São Paulo State University (UNESP), Botucatu, Brazil; b Instituto Cohen, São Paulo, Brazil; c Orthopaedics and Traumatology Department, Federal University of São Paulo, São Paulo, Brazil; d Hospital Israelita Albert Einstein, São Paulo, Brazil

**Keywords:** Allogeneic adipose-derived mesenchymal stem cells, Articular cartilage repair, Osteochondral histopathology, Ovine pre-clinical model, Polydioxanone scaffold, Tissue engineering

## Abstract

**Introduction:**

Articular cartilage exhibits limited intrinsic regenerative capacity, making its repair a persistent clinical challenge. Polydioxanone (PDO) scaffolds offer biocompatibility, tunable degradation, and structural support, whereas adipose-derived mesenchymal stem cells (AD-MSCs) possess chondrogenic, immunomodulatory, and regenerative potential. This study evaluated the biocompatibility of the PDO scaffold *(Plenum Tissue Ortho)* and investigated its effectiveness in enhancing articular cartilage repair when combined with microfracture (MF) or allogeneic ovine adipose-derived mesenchymal stem cells (SadMSCs), in comparison with a collagen scaffold (COL), in a preclinical ovine model.

**Methods:**

Twenty-four adult sheep were randomly allocated into six groups. Full-thickness chondral defects were created in the weight-bearing region of the medial femoral condyle and treated according to each group: Control (no treatment), MF alone, MF/COL, MF/PDO, SadMSC/COL, or SadMSC/PDO. SadMSCs were isolated, expanded, and characterized for viability, immunophenotyping, and trilineage differentiation. The biocompatibility of the PDO scaffold was assessed by cell adhesion and proliferation using Live/Dead staining and scanning electron microscopy. After 26 weeks, articular cartilage repair was evaluated macroscopically following ICRS scoring guidelines, histologically using the modified O'Driscoll score, and immunohistochemically for COL I, COL II, COL X, TGF-β2, and TGF-β3.

**Results:**

PDO scaffolds supported the survival, adhesion, and proliferation of SadMSCs. Clinically, animals showed good recovery without major complications. At necropsy, no residual membranes, inflammation, or adverse reactions were detected. Macroscopically, the PDO/MF group presented the best repair scores, significantly superior to the Control, MF alone, MF/COL, SadMSC/COL, and SadMSC/PDO groups. Microscopically, SadMSC/PDO achieved the highest O'Driscoll and immunohistochemistry scores, with strong expression of COL II and TGF-β, along with reduced COL I and COL X, indicating hyaline-like cartilage formation. Multivariate analysis confirmed a strong positive correlation between COL II, TGF-β expression, and histological scores.

**Conclusion:**

PDO scaffolds demonstrated excellent biocompatibility and, when combined with SadMSCs, promoted superior cartilage repair compared with COL scaffolds or MF alone. While PDO/MF achieved the best macroscopic outcomes, SadMSC/PDO provided the most favorable microscopic and molecular findings, suggesting that allogeneic AdMSCs seeded on PDO scaffolds enhance hyaline-like cartilage formation. These results highlight PDO and AD-MSC-based constructs as a promising alternative for translational strategies in cartilage repair.

**The translational potential of this article:**

This study demonstrated that the combination of PDO scaffold and allogeneic AD-MSCs significantly enhances the quality of stifle cartilage repair in a sheep model. Furthermore, the validation of this approach in an optimal large-animal model underscores its translational readiness for clinical application.

## Graphical abstract

**Figure:**
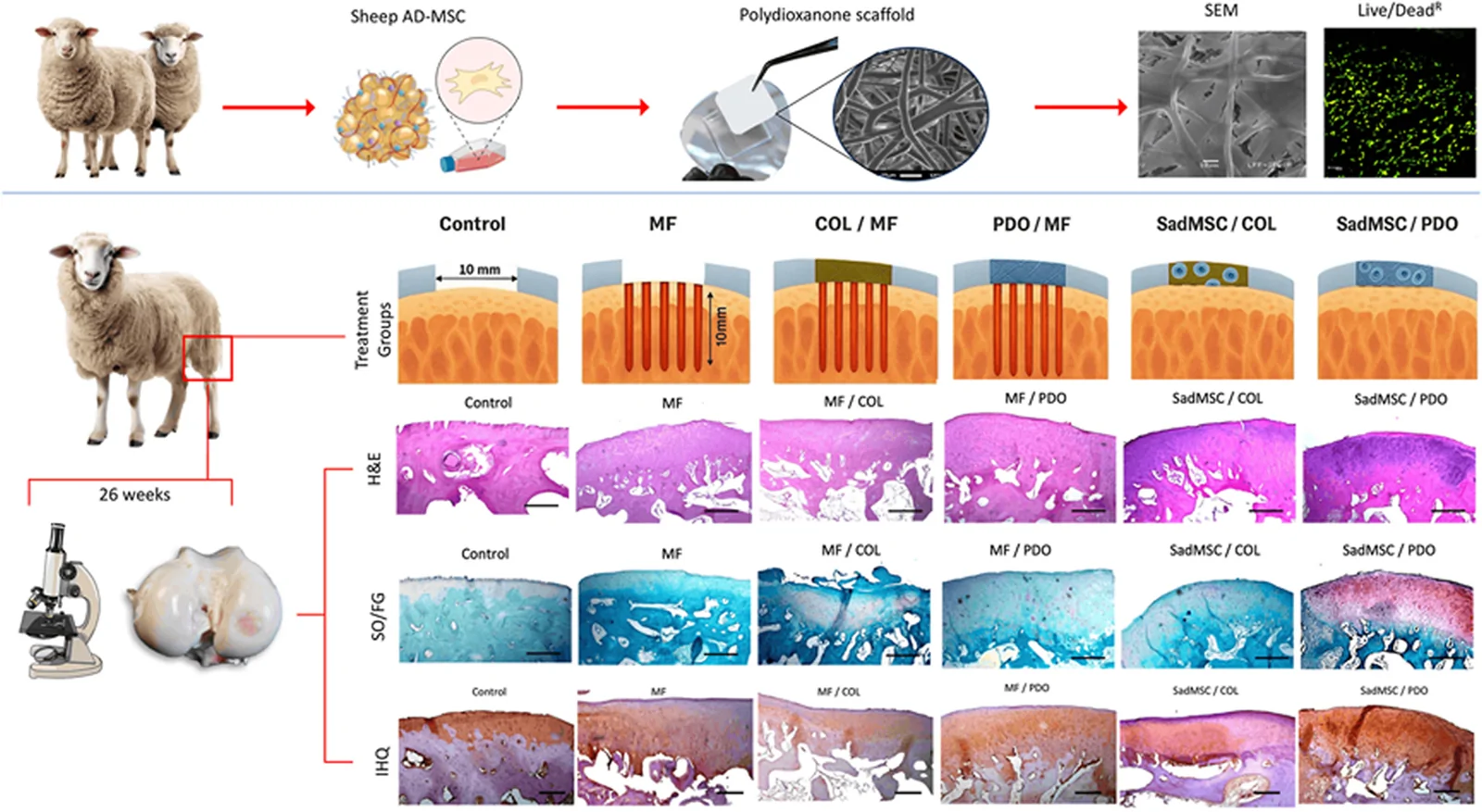

## 1 Introduction

Articular cartilage is a specialized connective tissue that covers the articulating surfaces of bones within synovial joints [1]. The articular chondrocyte is the single cell type in the cartilage and is responsible for the production and maintenance of the cartilage extracellular matrix (ECM) [2]. The ECM provides articular cartilage with its capacity to absorb and distribute compressive forces, ensuring the pain-free movement of the joints [1]^,^ [3]. This tissue lacks innervation, lymphatic vessels, and vasculature, resulting in a limited intrinsic capacity for repair. Furthermore, the cellular constituents of articular cartilage exhibit a low mitotic rate and are often metabolically quiescent, which slows ECM turnover and further hinders the tissue's regenerative capacity, contributing to the prolonged persistence of cartilage lesions [[1], [2], [3], [4]].

Despite the development of advanced therapeutic options, long-lasting cartilage repair strategies remain limited. This limitation underscores the clinical challenges in treating cartilage defects and degenerative joint diseases, necessitating the exploration of new materials and therapeutic approaches such as tissue engineering and regenerative medicine [5,6].

Polydiaxonone-based membranes have garnered attention in the field of tissue engineering and regenerative medicine for their potential applications in guided tissue repair. These membranes, composed of the biodegradable polymer polydioxanone (PDO), offer several advantageous properties such as flexibility, biocompatibility, and tunable degradation kinetics. The ability to control the degradation rate of PDO membranes enables tailored release kinetics of bioactive molecules, thereby promoting tissue regeneration while providing structural support to the defect site. Undergoing gradual hydrolytic degradation, polydioxanone generates non-toxic metabolites that are subsequently cleared from the body through renal excretion or pulmonary elimination [7]. The biodegradation time of the PDO membrane within the joint environment remains incompletely characterized; however, an estimated range can be inferred from the known degradation profile of PDO suture materials, which typically degrade and lose strength in 1-2 months and completely mass biodegrade at 6-12 months, depending on the local implantation conditions [8]. Additionally, PDO-based membranes have been shown to facilitate cell attachment, proliferation, and differentiation, thereby promoting the formation of functional tissue. These findings underscore the promising role of PDO-based membranes as scaffolds for tissue repair and regeneration in various clinical applications [7].

Since the discovery of mesenchymal stem cells (MSC) and the beginning of their clinical application, there has been a strong connection with orthopedic science [9,10]. Their multipotent nature, differentiation potential, immunomodulatory and paracrine properties have garnered increasing interest in the treatment of cartilage defects and osteoarthritis [10,11]. Moreover, their ease of harvesting and isolation from various sources, including adipose tissue [12], bone marrow [13] and umbilical cord blood [14], makes them accessible for orthopedic therapeutic applications [15].

Adipose-derived mesenchymal stem cells (AdMSCs) can be readily isolated in large quantities from the stromal vascular fraction of adipose tissue using minimally invasive harvesting procedures. These cells display robust clonal expansion under *in vitro* culture conditions and have demonstrated significant regenerative potential. Their well-documented chondrogenic differentiation potential makes them an attractive candidate for cartilage tissue engineering and repair strategies, offering the advantage of minimizing disruption to the patient's native cartilage homeostasis [16]. The use of allogeneic AdMSCs is enabled by their low immunogenicity and undifferentiated state, which render them less likely to trigger the host immune system. This strategy offers several advantages over autologous AdMSCs, including immediate availability (“off-the-shelf”), reduced cost due to large-scale production, and the procurement of cells from young and healthy donors, ensuring greater therapeutic potential. Moreover, it eliminates donor-site morbidity in the host and ensures the use of well-characterized MSCs, laboratory-certified for their immunological inertness, as evidenced by the absence of Major Histocompatibility Complex (MHC) expression [16,17].

This study aims to evaluate the biocompatibility and ability to support cell adhesion and proliferation of a PDO-based scaffold (*Plenum Tissue Ortho,* M3 Health Ind. Com. de Prod. Med., Odont. e Correlatos SA, Jundiaí, Sao Paulo, Brazil). Furthermore, the study investigates whether the PDO scaffold enhances the quality of articular cartilage repair, at the macroscopic, microscopic, and molecular levels, when used in combination with microfracture or directly seeded with allogenic AdMSCs, compared to the reference material and commercially available porcine collagen type I/III scaffold in a pre-clinical ovine model of full-thickness medial femur chondral defects and a 6-month follow-up period.

## 2 Material and methods

### 2.1 Animals

This study was approved by the Ethics Committee for the Use of Animals in Research at the Faculty of Veterinary Medicine and Animal Science of the São Paulo State University (UNESP), of the Botucatu campus (protocol n° 0068/2021-CEUA). Twenty-four mature female sheep (Ile de France) with a mean body weight of 59 kg (±10,2 kg) and ages between 4 and 6 years old were included. Clinical examination, lameness, and X-ray assessments were performed to ensure the healthy conditions of selected animals.

### 2.2 Sheep adipose-derived mesenchymal stem cells (isolation, cultivation)

Under surgical conditions, 3 g of subcutaneous adipose tissue were obtained from the tail base of two donors and placed in phosphate-buffered saline (PBS, Thermo-Fisher Scientific, Grand Island, NY, USA). The tissue was washed in PBS several times to remove blood clots, dried in sterile absorbent paper, and minced with a #24 scalpel blade. Tissue dissociation was made using 4 mg of type I collagenase (Sigma–Aldrich, St. Louis, MO, USA) diluted in Dulbecco's Modified Eagle Medium F12 GlutamaxTM (DMEM, Invitrogen, Grand Island, New York, USA) for each gram of adipose tissue. The content was homogenized and transferred to an incubator for 3 h at 37°C and 5% of CO_2_.

Collagenase was inactivated with the addition of DMEM F12 GlutamaxTM supplemented with 10% fetal bovine serum (FBS, Thermo Fisher Scientific, Grand Island, New York, USA), and the sample was centrifuged for 10 min at 460 g. After disposing of the supernatant, the sample was washed and centrifuged once again. The pellet of cells was diluted in 2 ml of culture medium (DMEM F12 Glutamax + 10% FBS + 1% penicillin, streptomycin, and amphotericin B solution - Antibiotic-Antimycotic, Thermo Fisher Scientific, Grand Island, New York, USA) and seeded in 25 cm^2^ culture bottles, changing the culture medium every 48h. Upon reaching a confluence of 80% or greater, cell adhesions were disrupted using TrypLE (Thermo Fisher Scientific, Grand Island, New York, USA). The tube content was centrifuged, and the pellet was resuspended in 2 ml of culture medium and seeded in 75 cm^2^ culture bottles (first passage or P1) and 175 cm^2^ bottles (second passage or P2 and third passage or P3). Cell viability was evaluated after each cell passage using the trypan blue exclusion assay in combination with an automated cell counter (Countess II FL Automated Cell Counter™, Thermo Fisher Scientific, Grand Island, NY, USA).

Cell characterization and pluripotency tests were performed by morphological evaluation and trilineage differentiation assays (chondrogenic, osteogenic, and adipogenic) [18]. For adipogenic and osteogenic differentiation, cell cultures in the third passage received medium StemProTM Adipogenesis Differentiation Kit (Gibco, Grand Island, NY, USA), whereas osteogenic differentiation was performed using StemProTM Osteogenesis Differentiation Kit (Gibco, Grand Island, NY, USA). The culture medium was changed every 48 h for 14 days. Cells were fixed in formaldehyde, and the osteogenic/control groups were stained with Alizarin Red, whereas the adipogenic/control groups were stained with Oil Red O. A Chondrogenic differentiation assay was made in biomass culture in a 15 ml conical centrifuge tube using StemProTM Chondrogenesis Differentiation Kit (Gibco, Grand Island, NY, USA) for 21 days. The culture was fixed and submitted to histopathologic evaluation using Toluidine Blue and Alcian Blue staining.

Immunophenotyping was conducted employing a real-time polymerase chain reaction (qPCR) assay, as previously delineated [19,20]. The cell lysis, mRNA extraction, cDNA synthesis, and qPCR were performed according to the previous literature [21]. Briefly, mRNA was extracted using the RecoverAll™ Total Nucleic Acid Kit (Ambion, Life Technologies, MA, USA), and mRNA quantity was evaluated using a spectrophotometer (NanoDrop ND-1000, Thermo Fisher, Waltham, MA, USA). The primer sequences of selected genes are described in Table 1. The GAPDH was used as an endogenous gene and target genes related to MSC pluripotency, OCT-4, SOX-2, and NANOG, as well as transcription factors of MSC surface markers CD73, CD90, CD105, CD34, and CD45. qPCR genes were conducted in a total volume of 10 μL containing Power SYBR Green PCR Master Mix (Applied Biosystems; Foster City, CA, USA), 1 μL of cDNA (1:10), and 0.3 μM of each primer. The reactions were performed in triplicate in 288-well plates using QuantStudio 12K Flex Thermal Cycler equipment (Applied Biosystems, Foster City, CA, USA). The PCR product specificity was determined by the dissociation curve for all experiments. Relative gene expression was quantified using the 2−ΔΔCT method.

**Table 1:** Sequences of primer pairs, accession numbers and sizes of PCR products used for PCR analysis.

N	Gene	Primer sequence	Accession number	Amplicon size	Ref
1	GAPDH	F - CTTCATTGACCTTCACTACATGG	NM_001190390.1	365	19
		R - TGCAGGAGGCATTGCTGACAA			
2	OCT-4	F - GATCGGGCCGGGGGTTGTGC	XM_004018968.1	235	
		R -TCGGCTCCAGCTTCTCCTTGTCCA			
3	SOX-2	F- CATGAACGGCTCGCCCACCTACAG	XM_004003838.1	267	
		R -TCTCCCCCGCCCCCTCCAGTTCAC			
4	NANOG	F - TTCCTTCCTCCATGGATCTG	FJ970651.1	315	
		R - ACCACTGGTTGCTCCAAGAC			
5	CD73^+^	F - TGGTCCAGGCCTATGCTTTTG	BC114093	115	18
		R - GGGATGCTGCTGTTGAGAAGAA			
6	CD 90^+^	F - CAGAATACAGCTCCCGAACCAA	BC104530	96	
		R - CACGTGTAGATCCCCTCATCCTT			
7	CD105^+^	F - CGGACAGTGACCGTGAAGTTG	NM_001076397	115	
		R -TGTTGTGGTTGGCCTCGATTA			
8	CD34^−^	F – TGGGCATCGAGGACATCTCT	AB021662	107	
		R - GATCAAGATGGCCAGCAGGAT			
9	CD45^−^	F – CCTGGACACCACCTCAAAGCT	NM_001206523	101	
		R-TCCGTCCTGGGTTTTATCCTG			

### 2.3 PDO and COL scaffolds porosimetry

The mercury porosimetry technique was used to determine the total porosity and the mean pore diameter. The test was carried out by the laboratory AFINKO Soluções em Polímeros Ltda (São Carlos, SP, Brazil).

The real density of the samples was determined using a helium pycnometer. Briefly, samples were weighed in a crucible of known volume and analyzed under helium atmosphere to determine the true volume. Density was calculated as the ratio of sample mass to measured volume. Porosity was evaluated by mercury intrusion porosimetry. Samples were weighed and placed in a graduated tube, followed by vacuum application to remove air from the pores. The tube was then filled with mercury and transferred to the analysis chamber. Pressure was applied using isopropyl alcohol to force mercury intrusion into the pore structure. The relationship between applied pressure and intruded mercury volume was recorded.

Total porosity (%) was calculated using the following equation:

$$\text{Total}\;\text{porosity}\;\left(\%\right)=V_{\max}\;/\;\left[V_{\max}+\left(m/\rho \right)\right]$$

where V_max_ is the maximum volume of mercury intruded, m is the sample mass, and ρ is the real density.

Porosity (%) and average pore diameter (μm) were obtained from the intrusion data. The analysis range of the equipment covered pore diameters from 0.35 μm to 100 μm.

### 2.4 Live/dead cell staining and scanning electron microscopy

The PDO scaffold (Plenum Tissue Ortho) material characterization has been previously performed by Saska et al. [7]. The seeding process and cell viability were confirmed with the Live/Dead cell staining. The staining process and Confocal Laser Scanning (CLS) evaluation were carried out 48 h after seeding the membrane with SadMSC. After washing the PDO membranes, a solution of 0.1 nmol calcein AM (C1359 Sigma–Aldrich) diluted in DMEM F-12 culture medium was added until the membranes were covered. The membranes were then incubated in the dark at 37°C for 15 min and washed once with PBS. Then, 1.0 μL/mL propidium iodide (Sigma–Aldrich) diluted in PBS was added for 10 min. The structures were immediately assessed by CLS (LEICA, TCS SP8) in a range of λ = 494-517 nm for calcein (green fluorescence) and λ = 535-617 nm for propidium iodide (red fluorescence).

For the Scanning Electron Microscopy (SEM), the PDO scaffold was seeded with 3 × 10^6^ of SadMSC in the same manner as previously described, and incubated for 11 days, with medium exchange every other day. Membranes were fixed in 2.5% glutaraldehyde and then metallized with gold deposition for the morphology evaluation by SEM (SEM - 6460LV, JEOL, Akshina, Japan).

### 2.5 PDO and COL scaffolds seeding with SadMSC

Forty-eight hours before the membrane surgical implantation, 3 × 10^6^ of SadMSC in the third passage were concentrated in 0.5 ml of DMEM F12 Glutamax™ (Thermo Fisher Scientific, Grand Island, New York, USA) and administered over a previously trimmed 10 mm diameter PDO and COL membrane. Due to the double layer, the COL membrane was placed backward for seeding on the rough and porous bottom surface. Subsequently, the seeded membranes were individually incubated in 35 mm culture plates. The membrane seeding process was conducted for 1 h prior to the immersion in 10 mL of DMEM-F12, which had been supplemented with 10% FBS (Sigma–Aldrich). This procedure was undertaken 48 h before the surgical application and provided the cell adaptation to the three-dimensional structure of the scaffolds [7].

### 2.6 Surgical procedure and establishment of cartilage defect

The sheep were deprived of food 24 h before surgery and provided water *ad libitum*. Prior to surgery, they were sedated with 0.3 mg/kg of Xylazine hydrochloride 2% administered intramuscularly (IM). The induction of anesthesia was achieved by the intravenous (IV) administration of 0.5 mg/kg of diazepam and 5 mg/kg of ketamine. After loss of consciousness, the sheep were positioned in sternal recumbence, skin clipped, and antiseptically prepared for a lumbosacral epidural anesthesia using 1 mg/kg of Lidocaine hydrochloride 2% and 0.2 mg/kg of Morphine sulfate injection. After the endotracheal intubation, anesthesia was maintained with 2.5% isoflurane mixed with 100% oxygen.

**Fig. 1:**
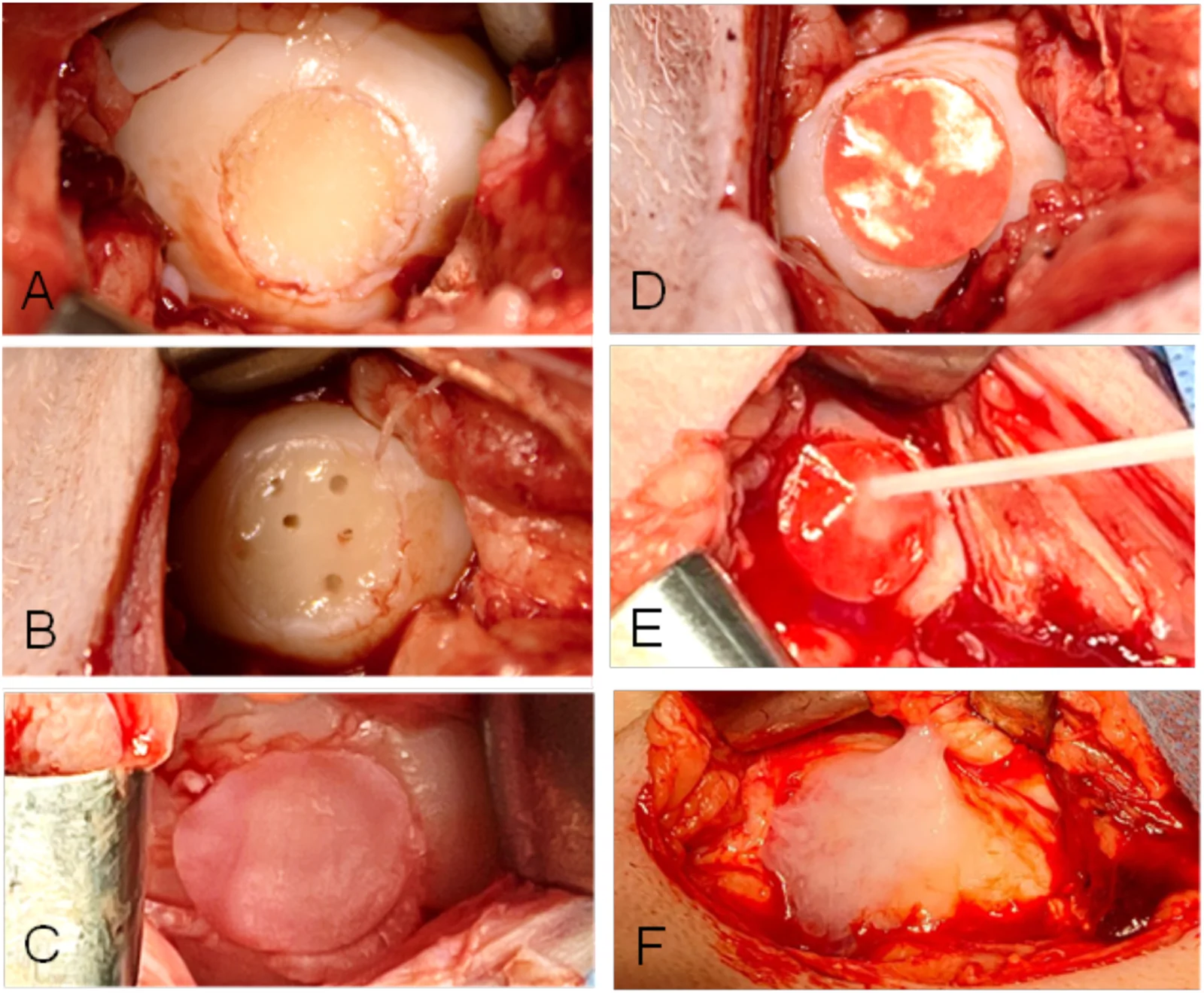
Images of the articular cartilage defect designed in the medial femur condyle, followed by the application of the treatment for each respective group. **A**. Chondral lesion measuring 10 mm in diameter. **B**. Chondral lesion combined with 5 microfracture, each measuring 1.5 mm in diameter within the subchondral bone. **C**. Application of COL membrane over the osteochondral and chondral lesion, representing COL/MF and SadMSC/COL groups. **D**. Application of PDO membrane over the osteochondral and chondral lesion representing PDO/MF and SadMSC/PDO groups. **E**. Application of Tissel® fibrin glue covering the chondral and osteochondral lesion and the membrane. **F**. Appearance of the fibrin sealant at the end of the gelation process.

**Fig. 2:**
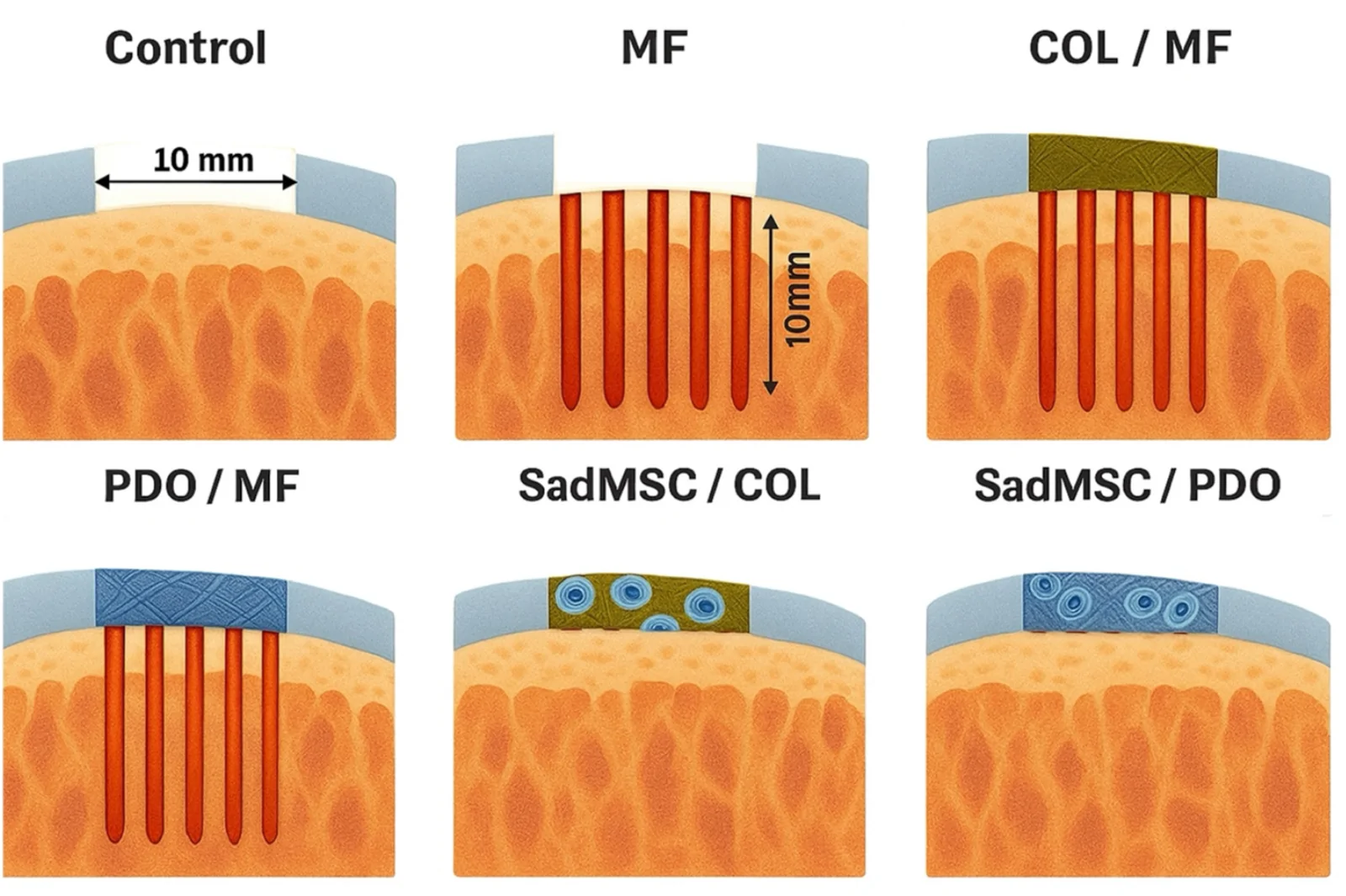
Experimental design of study groups and surgical treatments applied to the medial femoral condyle in sheep.

As soon as the anesthesia stabilized, the sheep were positioned in dorsal recumbency. Both stifles were clipped, disinfected, and approached for surgery. The surgical procedure began with a 5-cm skin incision made dorsomedial and parallel to the patellar tendon. Through blunt dissection, the femorotibial joint capsule was exposed, and arthrotomy was performed; the medial condyle of the femur was visualized, aided by flexion and external rotation of the tibia. A small amount of Hofa fat was removed to improve the medial femoral condyle exposition. Using a skin biopsy punch, a standard 10 mm diameter was delineated in the flat area of the weight-bearing center of the medial femur condyle. Full-thickness articular cartilage was excised with a fenestrated curette down to the calcified cartilage layer, ensuring preservation of the subchondral bone plate. Subchondral bleeding was not stimulated (Fig. 1A). According to each group, the treatment was applied ( see Fig. 2). The articular capsule, fascia, subcutaneous tissue, and skin were sutured in separate layers.

### 2.7 Experimental group design

A total of twenty-four sheep were assigned into six experimental groups, with eight stifles per group. Bilateral chondral defects were surgically created in the weight-bearing center of the medial femoral condyles, and the respective treatments were applied simultaneously. The determination of the experimental groups was based on the surgical techniques and their combinations employed in the treatment of focal cartilage lesions in the human knee. Random allocation was employed for all groups, except for the SadMSC-seeded scaffold groups. In these cases, each of the stifles from the same sheep received one COL and one PDO scaffold seeded with SadMSCs, in order to avoid contralateral interference through potential systemic MSC migration. ***Control group*** (Control): Following defect creation and skin closure, 3 mL of sterile saline solution (NaCl 0.9%) was intra-articularly injected. **Microfracture group (MF)**: Five perforations were made within the chondral defect, using a 1.5 mm Kirschner wire, to a depth of 10 mm. Subchondral bone penetration was confirmed by bone marrow bleeding (Fig. 1B). ***Microfracture + Collagen scaffold group (COL/MF) and Microfracture + Polydioxanone scaffold group* (PDO/MF):** After performing the same microfracture procedure, the defect was covered with either a COL (Fig. 1C) or PDO (Fig. 1D) scaffold, each trimmed to 10 mm in diameter and 0.5 mm in thickness. Scaffold fixation was achieved with 4 mL of fibrin sealant (Baxter, Vienna, Austria), applied over the scaffold and surrounding cartilage (Fig. 1E). Membrane attachment and stability were assessed after 6 min, as recommended by the manufacturer (Fig. 1F). **SadMSC-seeded Collagen scaffold group (SadMSC/COL) and SadMSC-seeded Polydioxanone scaffold group (SadMSC/PDO):** A chondral defect identical to the Control group was created, and coated with either a COL or PDO scaffold (10 mm in diameter, 0.5 mm in thickness) previously seeded with 3 × 10^6^ allogenic SadMSCs. Scaffold fixation was performed using 4 mL of fibrin sealant (Baxter, Vienna, Austria), as described for the MF + Scaffold groups (Fig. 1E and F).

### 2.8 Postoperative management

Recovery from anesthesia was carried out alone in a cage measuring 2 × 1 m until the ability to stand and walk was fully restored. Then, the sheep were transferred to 5 × 5 m stalls in groups of 6. The sheep were administered 2.2 mg/kg of ceftiofur and 20 mg/kg of dipyrone, IV for 10 days, as well as 0.5 mg of maxican 2%, IV for seven days. The surgical wound was cleaned and sprayed with a 2% iodine solution twice a day. Skin sutures were removed 14 days postoperatively. Four weeks after surgery, the sheep started the physical therapy protocol with 10 min of free walking and grazing in a 10 × 20 m paddock. The amount of time they spent grazing increased by 10 min each week until, at week 15 post-surgery, they were sent out to graze full-time.

### 2.9 Euthanasia

At week 26, all sheep underwent humane euthanasia. They were initially sedated with 0.3 mg/kg of 2% xylazine hydrochloride administered IM. Once sedation signs were observed, an overdose of 4 mg/kg of Ketamine was administered IV for anesthesia induction, followed by a hyper-concentrated potassium chloride solution, stopping the cardiac function.

Immediately after confirmation of death, both hind limbs were harvested. The joint soft tissues were examined for adhesions or signs of inflammation. The distal femur segment was dissected, removing the soft tissue and exposing the articular surface of both femoral condyles. These were then separated at the distal metaphysis and meticulously dissected.

### 2.10 Macroscopic assessment

Standardized photos and videos were taken of the distal femur segment, including the medial operated and lateral non-operated femoral condyle. Macroscopic cartilage repair scores, according to the International Cartilage Repair Society (ICRS; Brittberg & Winalski, 2003), were performed blindly by two experienced orthopedic doctors and one orthopedic veterinarian.

### 2.11 Microscopic analysis

The medial condyles were trimmed using an orthopedic sagittal saw and fixed in a neutral-buffered 10% formalin solution for a period of 48 h. The cartilage repair area was meticulously dissected into 4 x 4-cm pieces, ensuring a 1-cm tissue margin surrounding the lesion and 2 cm of subchondral bone. The samples were placed in a 5% nitric acid decalcification solution and replaced every three days until complete decalcification. Subsequently, the samples were dehydrated in increasing concentrations of ethyl alcohol, cleared with xylene, and embedded in molten paraffin wax. Following the preparation of the paraffin block, it was sectioned into 4 μm-thick slices using a microtome. Routine processing for histological examination in formalin-fixed, paraffin-embedded (FFPE) tissue was then carried out. For the microscopic exam, all of the histological sections were stained with hematoxylin and eosin (H&E), safranin O/Fast green, and picrosirius red. The assessment of the slides was conducted by two experienced veterinary pathologists who were unaware of the sample identity, following the fundamental principles of the modified O'Driscoll score [22]. Using polarized light microscopy, picrosirius red-stained samples were imaged to investigate collagen fiber alignment.

### 2.12 Immunohistochemistry

The tissue sections obtained from the FFPE blocks were placed on charged slides (Starfrost, Knittel, Bielefeld, Germany). For the antigen retrieval, the slides were incubated (37°C) in a pepsin solution (pH 1,8) for 30 min. Endogenous peroxidase was blocked by hydrogen peroxide 8% diluted in methyl alcohol for 20 min and treated with skim milk 8% for 40 min at room temperature. The sections were incubated overnight at 4°C with primary antibodies anti-COL-1 (COL1A1, 1:100; monoclonal ab6308; Abcam), anti-COL-2 (COL2A1, 1:200; monoclonal ab34712; Abcam), anti-COL-X (COL10A1, 1:100; polyclonal A6889; AbClonal), anti- TGF-β2 (1:100; polyclonal; 12HCLC, Invitrogen), and anti-TGF-β3 proprotein (1:500; polyclonal - ab227711, Abcam). A polymer detection system (Envision, Dako, Carpinteria, CA, USA) was applied as a secondary antibody, and immunoreactive cells were visualized by colorimetric detection (3,3ʹ-diaminobenzidine). The sections were counterstained with Harris hematoxylin (Dinamica, Diadema, Brazil) in order to enhance the visualization of cellular morphology and tissue organization. The non-operated lateral femur condyle was used as a positive and negative control for all antibodies.

The semiquantitative immunohistochemical scoring system proposed by Alcaide-Ruggiero et al. [23] was utilized to assess the immunostaining pattern and staining intensity of the images. In summary, this evaluation encompasses an analysis of the four distinct layers of cartilage and a subsequent comparison with the histology of healthy cartilage. Scores are assigned based on the number of layers that resemble natural cartilage, ranging from 0 (severely abnormal) to 3 (normal). The images were blindly graded by two veterinary pathologists and two orthopedic surgeons.

### 2.13 Statistical analysis

To analyze gene expression assays, ICRS macroscopic score and grading system, histological modified O'Driscoll score, and immunohistochemical score system, the data underwent a normality test (Shapiro–Wilk). Then, multiple comparisons were performed using One-way ANOVA (non-parametric) analysis of variance. A p-value of less than 0.05 was considered significant (GraphPad Prism v8.00; GraphPad Software, USA). In the description of the figures, the P values are understood to be: P ≤ 0.05*, P ≤ 0.01**, P ≤ 0.001***, and P ≤ 0.0001****. Absolute values are described in the body of the text.

The multivariate Spearman correlation test was used to assess associations among the variables macroscopy, histology, and immunohistochemistry (GraphPad Prism v8.00; GraphPad Software, USA).

## 3 Results

### 3.1 SadMSC characterization

**Fig. 3:**
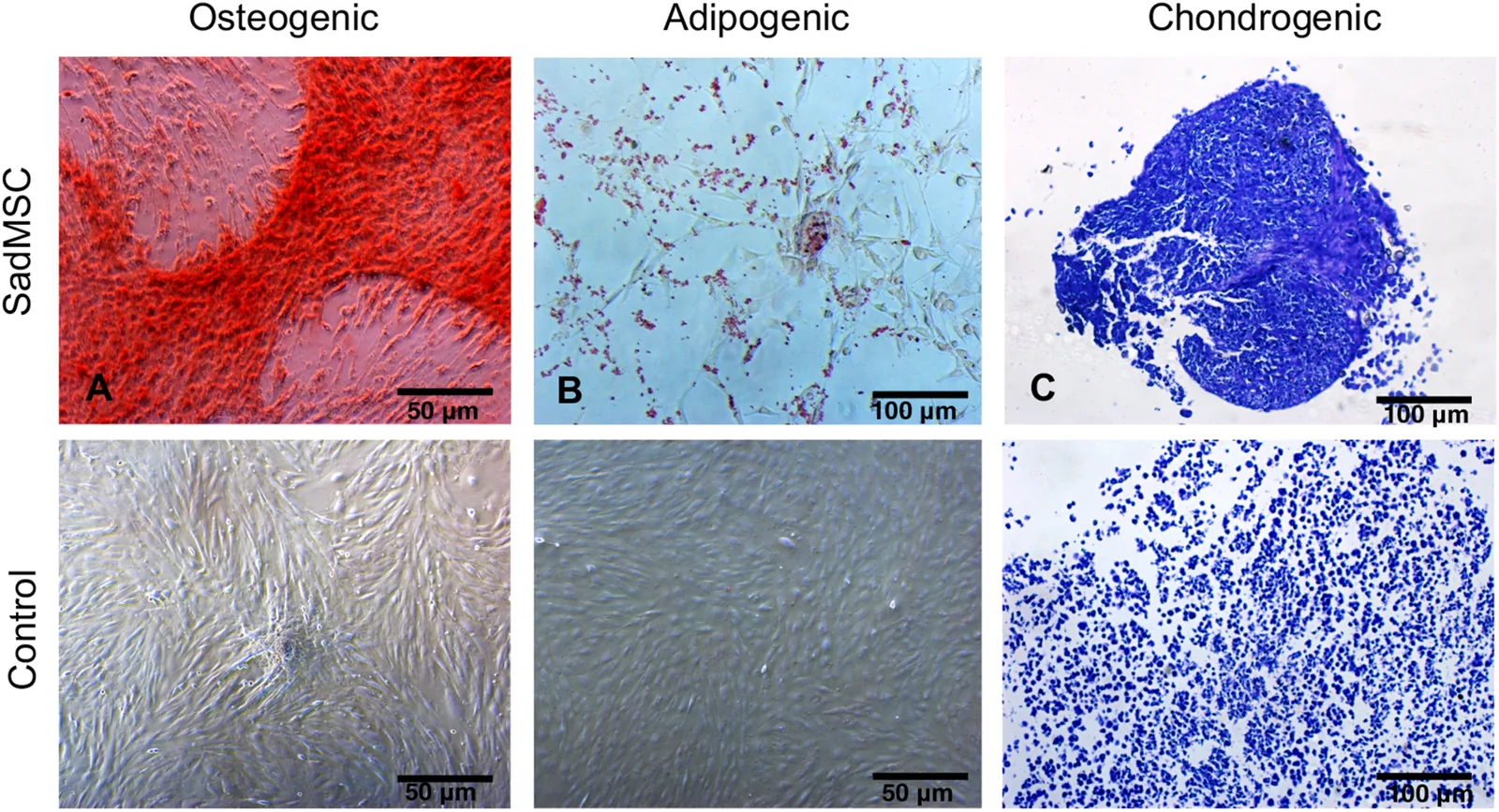
Trilineage differentiation of SadMSC. **A**. Osteogenic differentiation stained with Alizarin Red. **B**. Adipogenic differentiation stained with Oil Red. **C**. Chondrogenic differentiation stained with Toluidine Blue. The respective differentiation controls are located below.

Cells exhibited plastic adherence and clonal expansion capacity in standard culture conditions and were homogeneous in size and fibroblastoid-like morphology. Furthermore, the SadMSCs were capable of differentiating into the osteogenic, chondrogenic, and adipogenic lineages, confirmed by staining with Alizarin red, Toluidine blue, and Oil red, respectively (Fig. 3). Positive genic expression of surface markers CD 105, CD 73, and CD 90; negative expression of CD 45 due to not gene detection at 40 cycles of genic replication, and low expression of CD 34. Furthermore, the positive expression of the multipotent markers OCT-4, SOX-2, and NANOG strengthens the multipotent characteristics of the SadMSC.

### 3.2 PDO and COL scaffolds porosimetry

The PDO scaffold exhibited higher porosity (81.51%) compared to the COL scaffold (68.28%). In contrast, the COL scaffold showed a slightly larger average pore diameter (5.00 μm) than the PDO scaffold (4.37 μm). Overall, these results indicate that while PDO scaffolds present a more porous structure, collagen scaffolds are characterized by marginally larger pore sizes.

### 3.3 PDO scaffold seeding and cytocompatibility

**Fig. 4:**
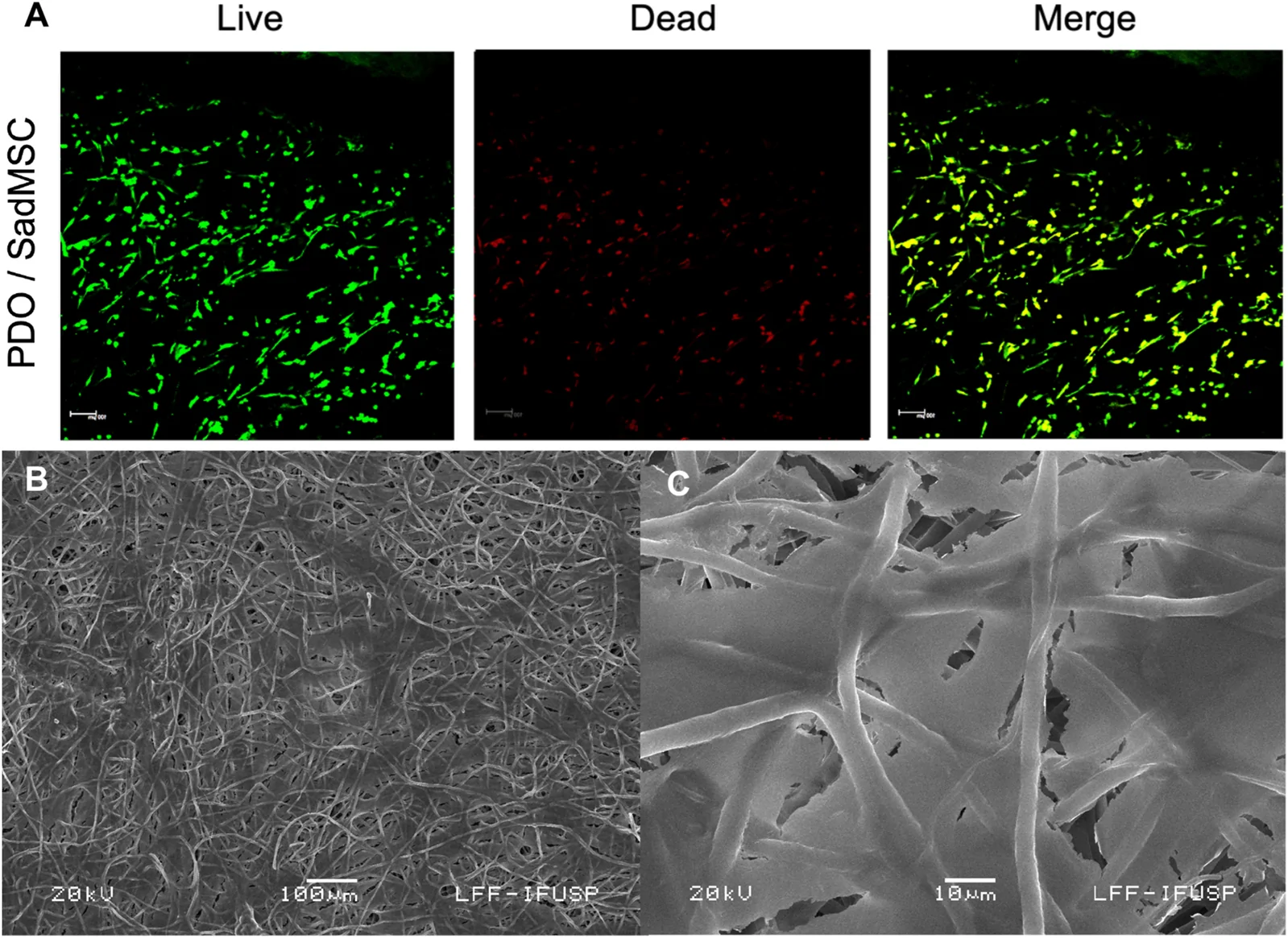
The biocompatibility and arrangement of SadMSC on the surface and inside the PDO membrane. **A.** The Live/Dead cell staining analyses were performed to evaluate the PDO scaffold after the 48-h seeding process. The SadMSC images were captured the distribution of live (green) and dead (red) cells. **B**. In scanning electron micrographs, the 11-day culture of SadMSC on PDO scaffold, exhibiting the production of ECM, an elongated shape coating the surface and interfilament spaces throughout PDO fibers. SEM 200x, 100 μm scale bar. **C**. SEM 2.000x, 10 μm scale bar.

The capacity of the PDO scaffold to preserve viability and facilitate integration with the SadMSCs was evaluated through the Live/Dead cell staining, in which the cells were seeded on the PDO membrane for 48 h. The cells were stained with a high concentration of calcein, which produced green fluorescence, indicating high viability. In contrast, there were few markings of propidium iodide, which exhibited red fluorescence and thus indicated cell death. Furthermore, the cells exhibited a fibroblast-like morphology, indicating an attachment process and adaptation to the scaffold filaments (Fig. 4A). A detailed evaluation of the SEM results revealed that the SadMSC produced ECM and formed continuous layers coating both the scaffold surface and interfilament spaces. The cells exhibited robust adherence to the PDO scaffold filaments, proliferation capacity, and a suitable distribution throughout the scaffold 11 days after seeding in the PDO scaffold (Fig. 4B and C).

### 3.4 Clinical observations

The ovine subjects demonstrated a positive recovery profile, with no adverse events, restriction of joint mobility, or ankylosis observed in the postoperative period. All knees exhibited a slight degree of effusion without lameness associated; nevertheless, complete resolution of the joint effusion and skin surgical incision healing was noted on day 21 post-surgery. One sheep from the PDO/MF group exhibited a progression from mild to severe effusion and lameness grade 4/6 [24] on the fifth postoperative day. The condition was managed by synovial fluid aspiration, followed by intra-articular administration of 100 mg amikacin sulfate, resulting in complete recovery by day 21, as evidenced by the absence of swelling and lameness.

### 3.5 Macroscopic evaluation

**Fig. 5:**
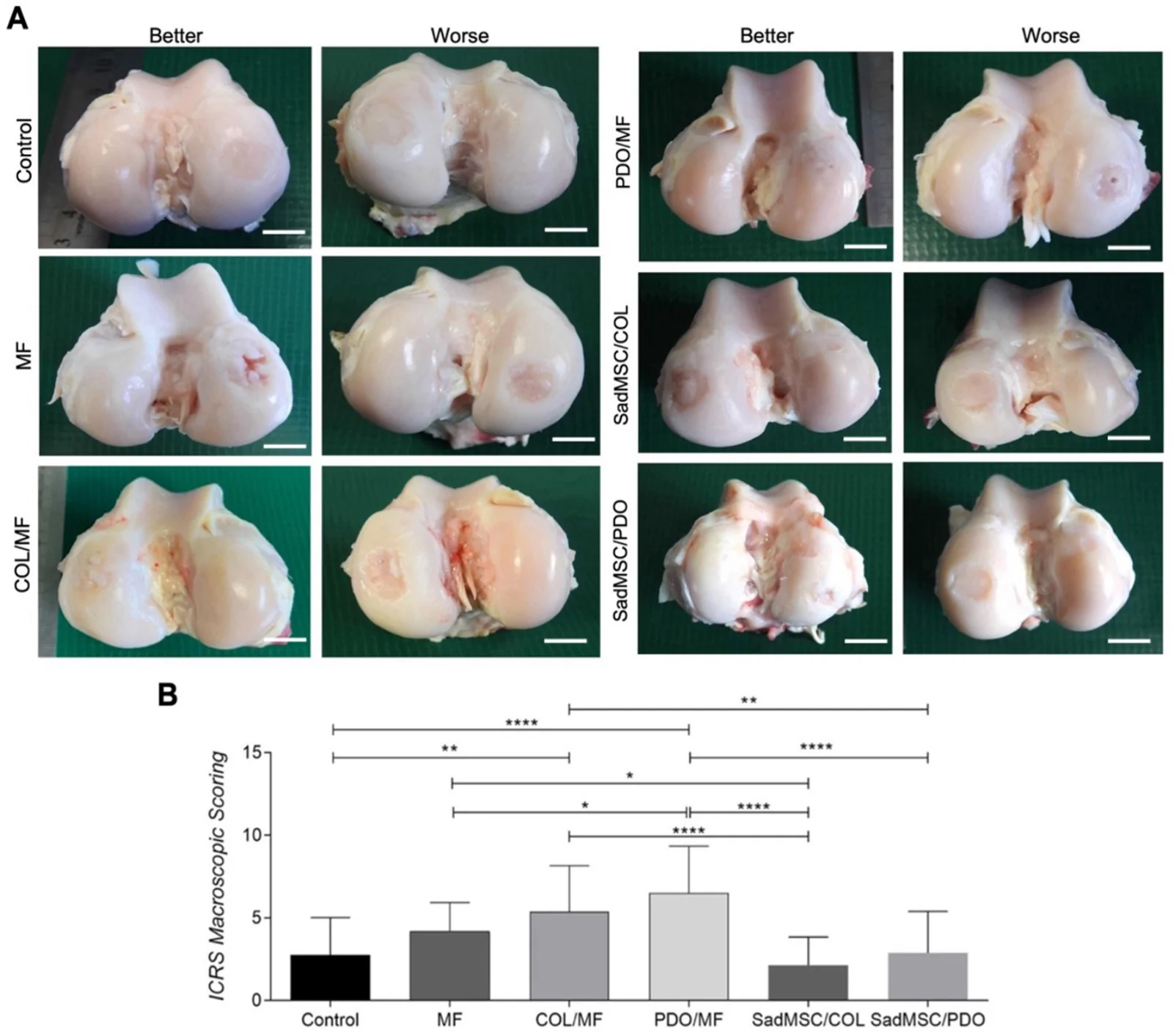
Macroscopic appearance of the cartilage repair 26 weeks post-surgery. **A.** Representative macroscopic images of the better and worse score according to the ICRS macroscopic assessment. **B.** ICRS scores. The group PDO/MF showed the higher ICRS score, statistically superior to Control, SadMSC/COL and SadMSC/PDO (≤0.0001): p ≤ 0.05*, p ≤ 0.01**, p ≤ 0.001***, and p ≤ 0.0001****.

Twenty-six weeks after surgery, the stifle joints of the sheep exhibited no synovial effusion and normal ranges of flexion and extension. Followed by joint dissection, no signs of infection or inflammation were observed in the joint capsule, synovial membrane, or fluid. A comprehensive evaluation revealed the absence of residual materials from the COL or PDO scaffolds, as well as from the fibrin sealant, in the joint space or in the repaired tissue. The macroscopic cartilage repair was evaluated using the ICRS macroscopic score and grading system. Better and worse images, according to the macroscopic scores, are displayed in Fig. 5A. The Control group showed minimal reparative ability, with the chondral defect shallowly filled with transparent chondral scar tissue (CST) and no bonding with the adjacent cartilage edges. Two of the eight stifles showed progressive articular cartilage degradation and focal osteoarthritis. Results in the MF group were variable. Most of the CST deposition followed an island-like pattern, overlying and confined to the microfracture holes at the level of the subchondral bone. Additionally, there was a notable absence of bonding with the healthy surrounding cartilage. In certain instances, the microfracture procedure was kept uncovered by CST, and erosions in the subchondral bone were discernible. The COL/MF group also showed remarkable CST deposition confined to the MF site, although surrounded by a thin layer of translucent and with irregular surface CST. Few areas of bonding with healthy cartilage were observed. In the PDO/MF group, CST deposition began in the microfracture holes and coalesced to fill in the majority of the chondral defect. The appearance of the CST was whitish and dense, with a slightly uneven surface. The degree of filling varied from 50 to 90% of the total area and depth of the cartilage lesion, with sites of bonding with healthy cartilage, although none of the chondral repairs achieved complete integration. In the SadMSC/COL group, a thin and translucent layer of CST was noted immediately over the subchondral bone and covering the entire chondral defect, along with a white and dense reparative tissue deposition in the center of the defect. While in the SadMSC/PDO group, the chondral defect presented white CST, most remarkable at the borders, with gradually reducing density in the center of the lesion. A similar thickness of the repaired cartilage was noticed in the area of integration with the contiguous healthy cartilage. The CST formation apparently followed a centripetal migration of cartilage deposition.

It is noteworthy that progressive degeneration of the articular cartilage surrounding the defects, characterized macroscopically by surface fibrillation and irregularities, was evident in the Control, MF, MF/COL, and MF/PDO groups. In contrast, this degenerative process was not observed in the SadMSC/COL or SadMSC/PDO groups.

The results corresponding to the grading of the blinded macroscopic assessment of cartilage repair according to the ICRS system are illustrated in Fig. 5B. The best macroscopic repair of the chondral defect occurred in the PDO/MF group, with statistically higher scores than the Control (*p* < 0.0001****), SadMSC/COL (*p* < 0.0001****), SadMSC/PDO (*p* < 0.0001****), and MF (*p* < 0.05*) groups. The COL/MF group was superior to the Control group (*p* < 0.01**), SadMSC/COL (*p* < 0.0001****), and SadMSC/PDO (*p* < 0.01**). The MF group showed statistically higher scores than the SadMSC/COL group (*p* < 0.05*).

### 3.6 Histological analysis

H&E: The study revealed the absence of inflammation or reactive foreign-body giant-cell reactions, suggesting that the implantation of COL or the PDO scaffold and SadMSC did not induce such responses. The control group exhibited minimal repair of the cartilage defect, as evidenced by the absence of CST formation or bonding with the adjacent cartilage in any of the stifles in the group. The surface of the calcified cartilage layer exhibited irregularities and fissures extending into the subchondral bone plate. There was an absence or minimal repair in the cartilage defect where the tide-mark was preserved. In the MF group, CST formation was observed in seven of the eight stifles. The neocartilage tissue exhibited an irregular or fibrillated surface, and it was completely stained with eosin. In three of eight stifles, bonding of the CST with the adjacent healthy cartilage was observed histologically, and in one case, reorganization of the tidemark was identified. Additionally, the presence of fibrocartilaginous tissue with hypertrophic chondrocytes was observed along the trajectory of the microfractures within the subchondral bone (Fig. S1A and B). Furthermore, islands of chondrocytes were identified within the subchondral bone, encircled by a cartilaginous matrix that exhibited a positive reaction to hematoxylin staining. In the MF/COL group, the presence of CST was observed in seven out of eight stifles, with a regular surface in one of the knees, while the remaining knees exhibited a fibrillated surface. Fibrocartilage deposition at the level of the adjacent healthy cartilage was observed in 2/8 cases, with blood vessels present inside and bonding with healthy cartilage at one edge in 4/8 cases (Fig. S1C and D). The MF trajectory was filled with a fibrocartilaginous tissue, stained with eosin. Areas of endochondral ossification were identified within the microfracture holes in two of the eight stifles examined. One notable finding was the presence of an overlying extension of the perichondrium from the healthy cartilage on the area of repair. The MF/PDO group exhibited CST in seven of the eight knees, characterized by a smooth surface and the organization in layers consistent with normal cartilage structure. Union with the adjacent cartilage occurred at both edges in one knee, and was limited to just one edge in six, with predominance of eosin staining. The SadMSC/COL group demonstrated CST within the chondral defects in three of the eight knees evaluated. The CST had a smooth surface and a layered distribution of chondrocytes. Of the eight knees examined, two exhibited regions of bonding between the CST and the healthy cartilage edges, which exhibited slight hematoxylin staining within the territorial and interterritorial matrix of chondrocytes. The SadMSC/PDO group achieved the highest scores according to the modified O'Driscoll histological scoring system (Table 2), exhibiting the most hyaline-like cartilage repair characteristics. Bonding to adjacent cartilage in both lesion extremities was noticed in 4 samples. The CST was characterized by a smooth surface, the thickest and well-organized stratification of chondrocytes in layers compared with the other groups. Hematoxylin staining was observed in all layers of the CST, with greater staining intensity at the site of bonding with the healthy articular cartilage. In the groups SadMSC/COL and SadMSC/PDO, no degenerative signs or hyaline cartilage infiltration were noticed in the subchondral bone.

**Table 2:** Modified Histological O'Driscoll Score at 26 weeks postoperatively.

O'Driscoll Score	Groups					
	Control	MF	COL/MF	PDO/MF	COL/SadMSC	PDO/SadMSC
Hyaline cartilage (0-8)	1 (0-2)	1 (2-6)	0	1 (0-2)	0	1 (0-7)
Surface irregularity (0-2)	0 (0-1)	1 (0-2)	0 (0-1)	1 (0-2)	0 (0-1)	0 (0-1)
Structural integrity (0-2)	0 (0-1)	1 (0-2)	0	0 (0-1)	0 (0-1)	0 (0-1)
Thickness (0-2)	0	0 (0-2)	0	0	0	0 (0-1)
Bolding to adjacente cartilage (0-2)	0 (0-1)	0 (0-1)	1 (0-1)	1 (0-2)	0 (0-1)	1 (1-2)
Freedom from cellular changes of degeneration (0-2)	0	1 (0-2)	1 (0-1)	1 (0-1)	0 (0-1)	0 (0-1)
Freedon from degenerate changes in adjacent cartilage (0-2)	1 (0-1)	1 (0-2)	1 (0-2)	2 (1-2)	2 (1-2)	2 (1-2)
Reconstitution of subchondral bone (0-2)	0	1 (0-1)	0 (0-1)	1 (0-1)	1 (0-2)	1 (1)
Bolding of repair cartilage to the new subchondral bone (0-2)	0	1 (1-2)	0 (0-1)	0 (0-1)	0 (0-1)	0 (0-1)
Safranin O staining (0-2)	0	0	0	0	0	0 (0-2)
**Total (0-27)**	**2 (0-6)**	**7 (0-18)**	**2 (0-6)**	**7 (2-11)**	**3 (2-5)**	**10 (4-19)**

**Fig. 6:**
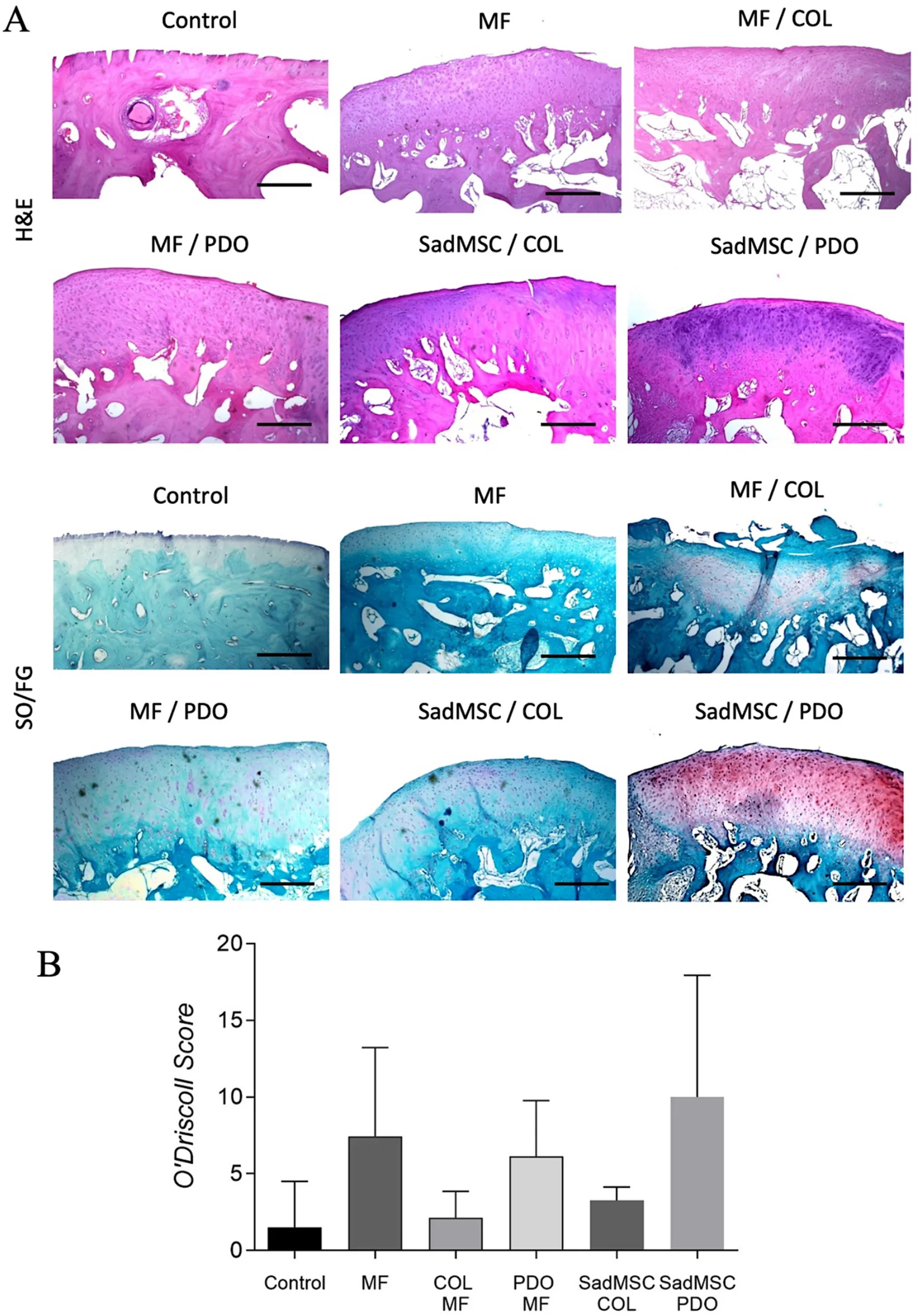
Histological assessment of the repaired articular cartilage. **A.** Group representative images for H&E and Safranin O/Fast Green (SO/FG) staining. **B.** Histological modified O'Driscoll scores expressed as mean and standard deviation (SD). Bar = 200 μm.

SO/FG Staining: In the control group, the CST stained positive for fast green, while Safranin O staining was absent in all eight defects analyzed. The MF group exhibited exclusive fast green staining in all CST subjects. Upon examination, the presence of Safranin O stained cartilage was observed within the microfracture tracts in the subchondral bone, accompanied by hypertrophic chondrocytes and endochondral ossification. In the MF/COL group, one of the CSTs exhibited slight Safranin O staining; however, it presented with a fibrillated surface and did not extend over the subchondral bone plate. In the MF/PDO group, mild Safranin O staining was noticed in four of the eight CST. Safranin O staining was more pronounced in the territorial matrix of grouped chondrocytes in the intermediate and deep layers of the repaired cartilage. Light Safranin O staining was observed in the territorial matrix of intermediate-layer chondrocytes in one sample from the SadMSC/COL group. The SadMSC/PDO group exhibited mild Safranin O staining in two stifles and strong staining in one. Intense Safranin O pigmentation was more evident at the edges of the cartilage repair and in the areas with chondrocyte clusters, while it was absent in the center of the lesion. An extension of cartilage and chondrocytes stained with safranin was noted into the subchondral bone, thereby maintaining a connection with the articular cartilage. Representative histological images stained with H&E and SO/FG are exhibited in Fig. 6A.

The modified O'Driscoll Score [22] assessed ten histological items of the cartilage repair site, and the results are summarized in Table 2. The maximum analysis score is 27 points. At 26 weeks after the surgical treatment application, the PDO/SadMSC group achieved the highest median O'Driscoll score, with a value of 10 (range: 4–19). Furthermore, the MF and PDO/MF groups demonstrated intermediate median scores of 7 (ranges: 0–18 and 2–11, respectively). The COL/SadMSC, COL/MF, and Control groups presented the lowest median scores, with values of 3 (range: 2–5), 2 (range: 0–6), and 2 (range: 0–6), respectively. Despite numerical differences among groups, no statistically significant differences were observed in the O'Driscoll scores across treatment groups (Fig. 6B).

**Fig. 7:**
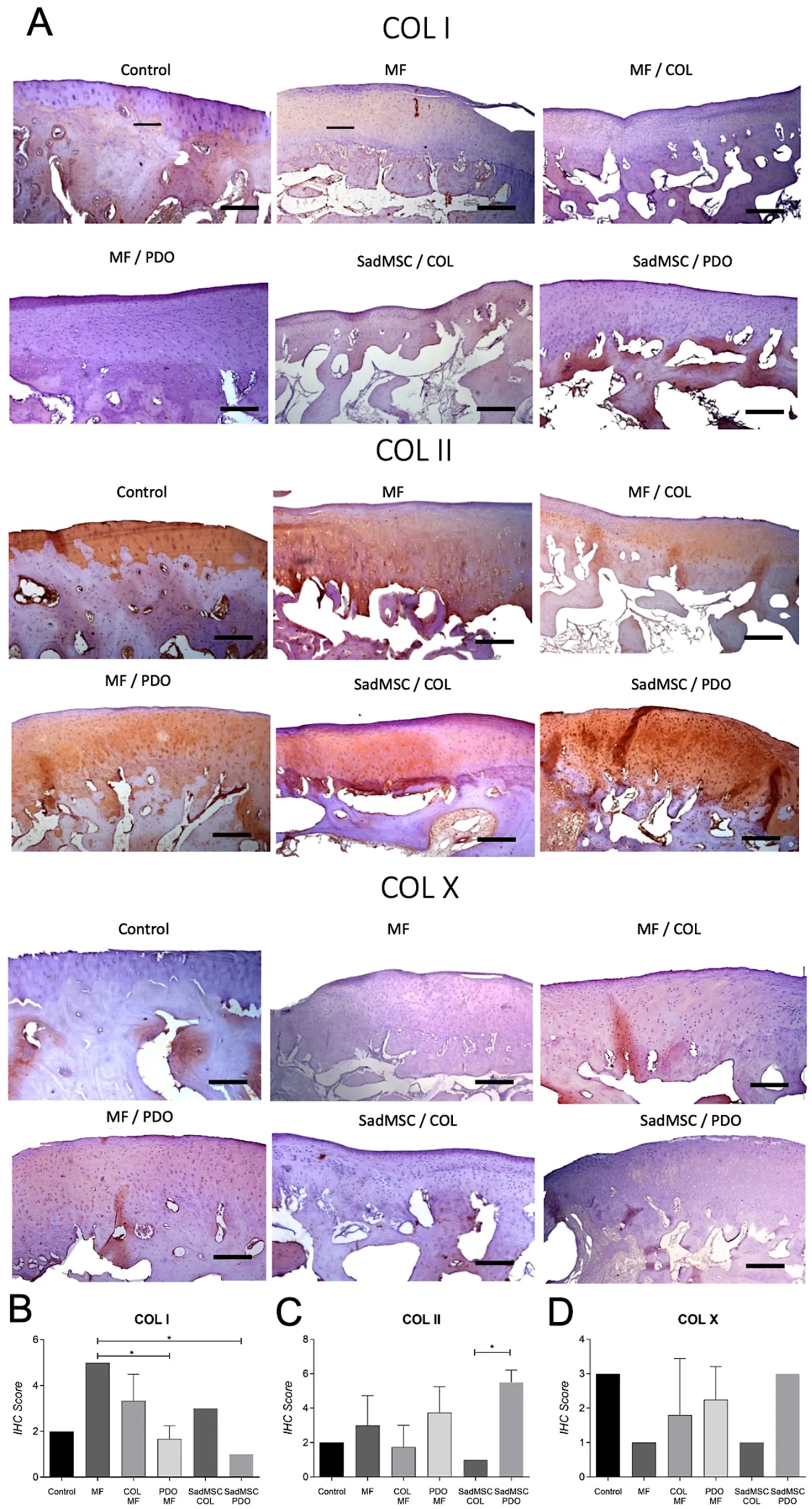
– Collagen immunohistochemical evaluation. **A**. Representative experimental group images of the collagen I, collagen II, and collagen X immunohistochemical evaluation. Semiquantitative immunohistochemical score for COL I (**B**), COL II (**C)**, COL X (**D**), TGFβ-2 (**E**), and TGFβ-3 (**F**). Values of p ≤ 0.05*, p ≤ 0.01**. DAB chromogen, Harris hematoxylin counterstain. Bar = 200 μm.

**Fig. 8:**
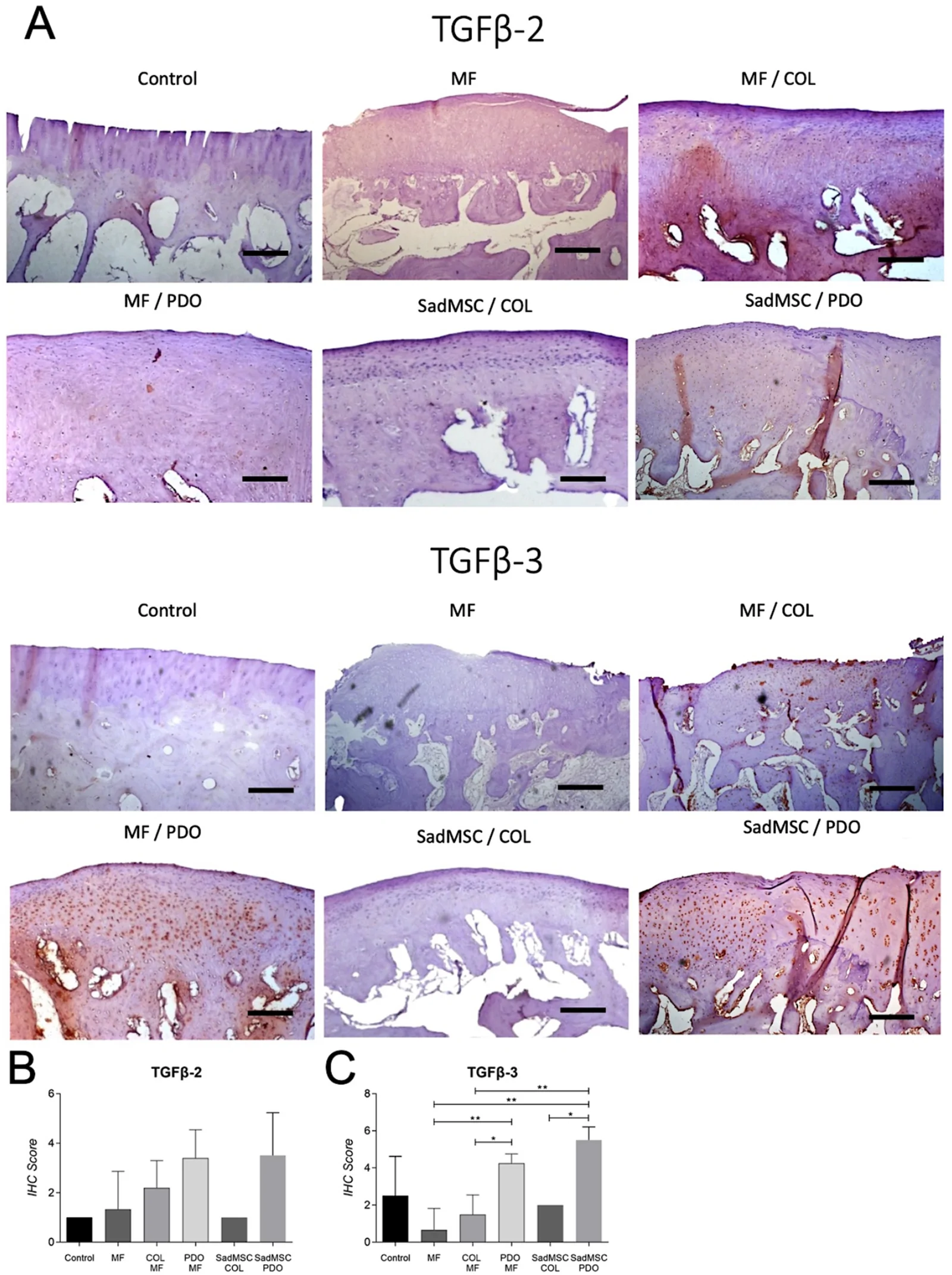
TGF-β immunohistochemical evaluation. **A.** Representative experimental group images of the TGFβ-2 and TGFβ-3 immunohistochemical evaluation. Semiquantitative immunohistochemical score for TGFβ-2 (**B**), and TGFβ-3 (**C**). Values of p ≤ 0.05*, p ≤ 0.01**. DAB chromogen, Harris hematoxylin counterstain. Bar = 200 μm.

IHC Analysis: The representative image of the immunostaining collagen labelling of each group is shown in Fig. 7A. **COL I**: The MF group showed significantly higher COL I expression than the Control (*p* < 0.05*) and SadMSC/PDO (*p* < 0.01**) groups (Fig. 7B). In the control group, the staining was exclusively present in the subchondral bone. The other groups, namely MF/COL, MF/PDO, SadMSC/COL, and SadMSCs/PDO, exhibited no positive staining in the repaired articular cartilage. **COL II**: The SadMSC/PDO group showed the highest COL II staining, statistically superior to the Control, COL/MF, and SadMSC/COL (*p* < 0.01**) groups (Fig. 7C). In the Control group, staining was restricted to the remaining calcified cartilage layer. In the MF group, COL II expression was detected in the intermediate and deep zones of the neoformed cartilage. In the MF/COL, MF/PDO, and SadMSC/COL groups, COL II staining was most evident in the deep layer of the repaired cartilage. In contrast, the SadMSC/PDO group demonstrated intense and homogeneous COL II immunostaining throughout all cartilage layers, with a significant upregulation in staining intensity within the chondrocytes of the regenerated cartilage. **COL X**: No statistically significant differences were noted among the groups (Fig. 7D). The higher staining intensity was observed in the chondrocytes and ECM of the transition and deep layers of the MF/COL group. In the MF/PDO group, staining was observed exclusively in the deep layer of the cells. In the SadMSC/PDO, positive staining was observed in the ECM of the intermediate layer of the cartilage. Representative image of the immunostaining TGF-β labelling of each group is shown in Fig. 8A. **TGFβ-2:** Groups COL/MF (*p* < 0.05*), PDO/MF, and SadMSC/PDO (*p <* 0.01**) had statistically superior staining compared to MF. Also, SadMSC/PDO (*p <* 0.05*) was statistically superior in staining compared to SadMSC/COL group (Fig. 8B). Both groups, MF/COL and MF/PDO demonstrated moderate TGFβ-2 expression in the ECM of the intermediate zone, along with positive labeling in chondrocytes located in the intermediate and deep layers. The SadMSC/PDO group showed moderate TGFβ-2 labeling in the ECM of the repaired cartilage and moderate expression in the chondrocytes of the intermediate and deep layers. **TGFβ-3**: The intracellular immunoreactivity is due to the detection of the precursor form of the protein. The SadMSC/PDO showed the strongest TGF-β3 expression, significantly higher than the MF (*p* < 0.05*) group (Fig. 8C). The MF/COL group displayed positive TGFβ-3 labeling in chondrocyte clusters located at the interface between native and repaired cartilage. The SadMSC/PDO group demonstrated the most robust TGFβ-3 labeling among all groups, with intense staining observed in chondrocytes across all layers of the repaired cartilage and within the ECM of the neoformed tissue. Strong immunolabeling was also detected in chondrocyte clusters at the interface between the native and regenerated cartilage.

**Fig. 9:**
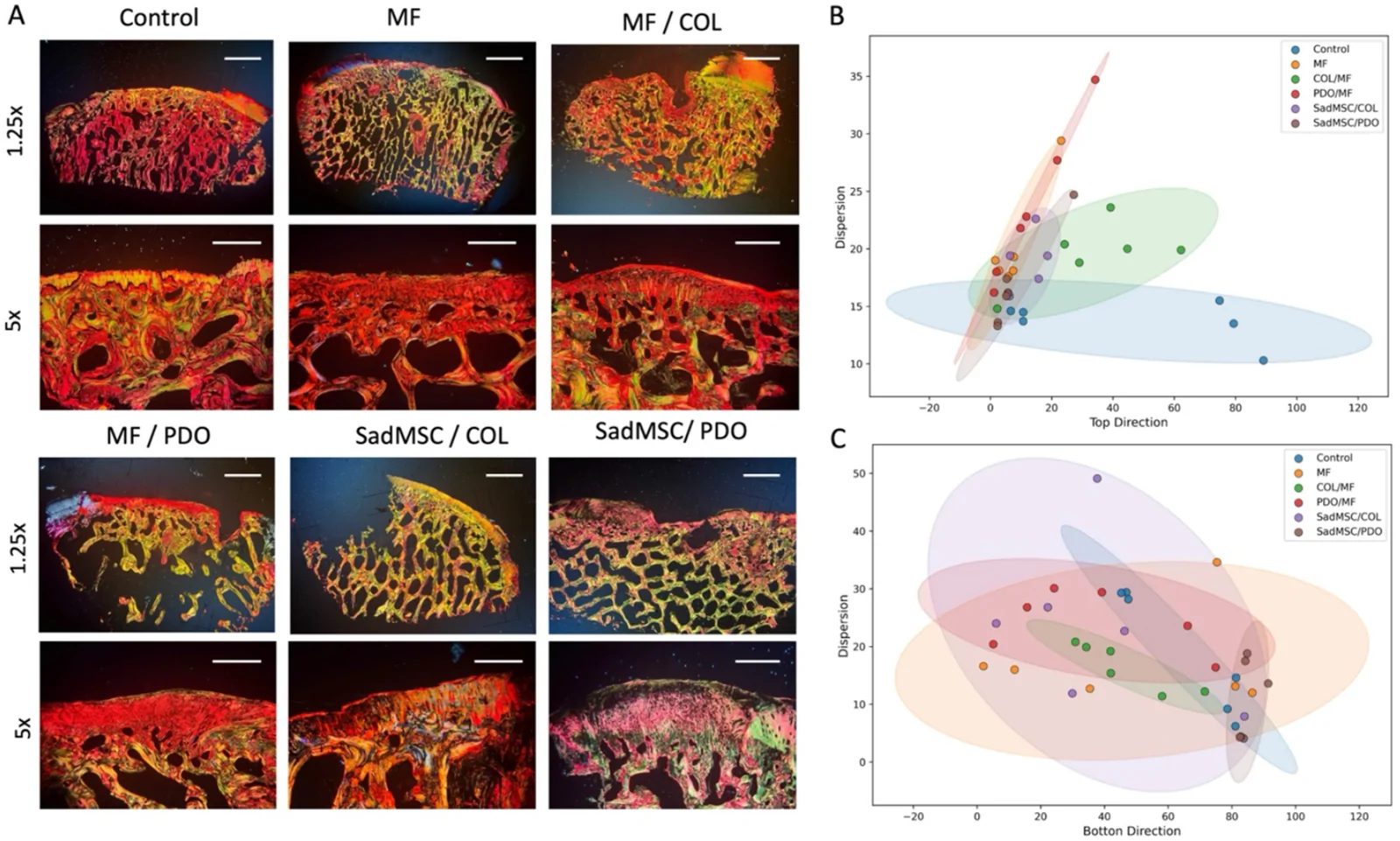
**–** Quantitative analysis of collagen fiber orientation in repaired cartilage tissue. (A) Representative images of each experimental group stained with Picrosirius Red and examined under polarized light microscopy at 1.25× and 5× magnifications. (B, C) Scatter plots depicting collagen fiber orientation in the superficial (top) and deep (bottom) zones of the repaired cartilage at 6 months postoperatively. Results are expressed as mean ± SD. Scale bars correspond to 100 μm in the 1.25× images and 200 μm in the 5× images.

Sirius-red staining: To assess collagen deposition and fiber orientation in the repaired cartilage of the medial femoral condyle, Sirius Red staining was performed and evaluated under polarized light microscopy. Images from each experimental group were analyzed using the Directionality plugin in ImageJ to quantify collagen fiber orientation and angular dispersion in both the superficial and deep zones of the repaired cartilage [25] (Fig. 9A). At the six-month postoperative time point, the control group exhibited no evidence of collagen deposition within the cartilage defect. Under polarized light, the lesion area displayed predominantly yellow and green birefringence. The MF group demonstrated partial defect filling by a thin layer of repair tissue, with predominantly red birefringence, suggesting the presence of more organized collagen fibers. In the MF/COL, MF/PDO, and SadMSC/COL groups, a moderate thickness of collagenous repair tissue was observed. The MF/COL group showed reddish birefringence interspersed with vertically oriented yellow streaks, indicating partial alignment of collagen fibers. In contrast, the MF/PDO group exhibited dense and uniformly red birefringence across all layers, consistent with a highly organized collagen network. The SadMSC/COL group displayed dense reddish birefringence in the superficial zone, while the deeper layers exhibited multidirectional striations with varying birefringence colors (red, orange, blue, and yellow), reflecting heterogeneous fiber. The SadMSC/PDO group showed repaired tissue with a thickness comparable to that of adjacent native cartilage. Under polarized light, the superficial zone exhibited green to light red birefringence, whereas the deeper layers predominantly displayed light red birefringence, suggesting improved structural organization relative to other groups. The orientation and angular dispersion of collagen fibers in both the superficial and deep zones of the SadMSC/PDO group more closely resembled those of native articular cartilage. Specifically, the superficial layer (top layer) exhibited a fiber orientation ranging from 0° to 17° (Fig. 9B), with an average angular dispersion of 16.8°. In the deep layer (bottom layer), the predominant fiber orientation ranged from 85° to 90° (Fig. 9C), with a mean angular dispersion of 10.4°, indicating a well-organized and zone-specific collagen architecture.

### 3.7 Multimodal evaluation of cartilage repair

**Fig. 10:**
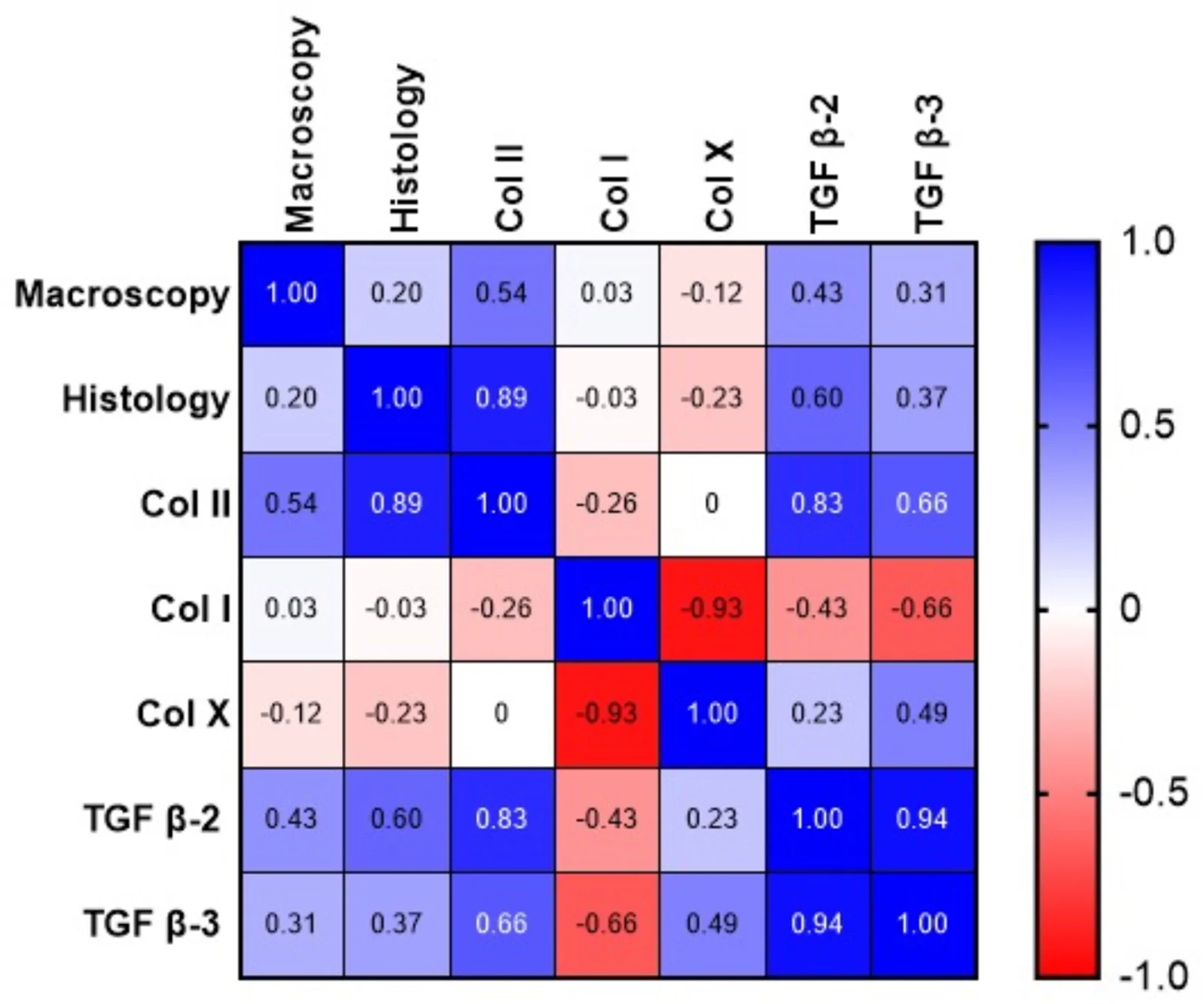
Multivariate analysis for ICRS macroscopic score, histological modified O'Driscoll score, and immunohistochemistry staining (COL II, COL I, COL X, TGF β-2, and TGF β-3) in the sheep model of cartilage repair.

Multivariate analysis included data from all 24 sheep with complete ICRS macroscopic score and grading system, histological modified O'Driscoll score, and immunohistochemistry results (Fig. 10). Strong positive correlations were observed between OHS and COL II (r = 0.89); COL II and TGF β2 (r = 0.83); COL II and TGF β3 (r = 0.66); TGF β2 and OHS (r = 0.60); and TGF β2 and TGF β3 (r = 0.94). Strong negative correlations were noted between COL I and COL X (r = 0.93), and COL I and TGF β-3 (r = 0.66). Moderate positive correlations were found between ICRS and COL II (r = 0.54); ICRS and TGF β-2 (r = 0.43); and COL X and TGF β-3 (r = 0.49). A moderate negative correlation was observed between COL I and TGF β-2 (r = 0.43).

## 4 Discussion

In this study, we first investigated the synthetic PDO membrane biocompatibility and its ability to provide physical and chemical support for the SadMSCs. Our findings, supported by previous studies [7], indicated that PDO scaffolds possess excellent cytocompatibility, preserving the SadMSC viability, while providing structural support for adhesion, proliferation, and facilitating cell migration. Subsequently, we assessed the effectiveness of the PDO scaffold, compared with a COL scaffold, combined with microfracture or allogenic SadMSCs for repairing full-thickness femoral condyle cartilage defects in a sheep model. At 26 weeks postoperatively, the PDO/MF group achieved the highest macroscopic scores, whereas the SadMSC/PDO group demonstrated the most favorable microscopic findings, including immunohistochemical scores. Although the modified O'Driscoll score did not demonstrate statistically significant differences, the SadMSC/PDO group consistently achieved the highest histological score, indicating a more favorable histological outcome. Notably, the MF group exhibited the highest immunostaining labeling for COL I in the repaired cartilage.

PDO is a synthetic, biodegradable polymer widely recognized for its excellent biocompatibility and adjustable degradation profile, making it a promising candidate for a range of tissue engineering applications [26,27]. Its application in bone [7,[28], [29], [30]] and neural [31] tissue regeneration has already been extensively explored. Although the ability of PDO scaffolds to induce *in vitro* chondrogenic differentiation of rabbit AdMSCs has already been demonstrated [7,32], the present study is, to our knowledge, the first to apply PDO scaffolds in a tissue-engineered construct combined with allogenic AdMSC specifically aimed at repairing articular cartilage defects. This novel application highlights the translational potential of PDO beyond *in vitro* settings.

Given the growing interest in the application of PDO-based materials for articular repair, a recent study evaluated the therapeutic potential of a soluble PDO viscosupplement in a rabbit model of collagenase-induced osteoarthritis [17]. Histopathological analyses demonstrated marked improvements in cartilage structure and matrix composition in the PDO-treated group. Molecular investigations revealed a significant downregulation of matrix metalloproteinases (MMP-3, MMP-9, and MMP-13) alongside upregulation of type II collagen and aggrecan. Additionally, ELISA measurements showed reductions in inflammatory markers, notably interleukin-1β and tumor necrosis factor-α. Taken together, these findings indicate that PDO articular injections may offer superior anti-inflammatory and cartilage-regenerative effects compared to traditional treatments, highlighting their promise as a novel viscosupplement for osteoarthritis therapy [17].

The cytocompatibility of the PDO scaffold was confirmed 48 h after being seeded with SAdMSCs, by the live/dead assay, and after 11 days through scanning electron microscopy (SEM). After 48 h of interaction, the cells exhibited a fibroblast-like morphology upon adhesion to the PDO scaffold fibers, maintaining high viability. By day 11 of 3D culture, extensive cell proliferation and infiltration were observed across the surface and within the filaments of the PDO scaffold. These findings are consistent with previous studies demonstrating the high biocompatibility of PDO scaffolds with both human and rabbit-derived AdMSCs [7,32]. Furthermore, in a recent study of soluble PDO cytocompatibility, rabbit chondrocyte viability remained stable after 24 h of culture in PDO solutions across ten different concentrations ranging from 0 to 30,000 mg/mL, indicating the low cytotoxicity of the material [17]. These findings reinforce the biosafety profile of PDO and its potential for applications in cartilage tissue engineering.

The biodegradability and degradation time of a scaffold should ideally match the healing rate of the tissue where it is implanted, thus maintaining the mechanical, noncytotoxic, and biologically inert properties [33]. In this regard, collagen-based membranes, although widely used in guided cartilage repair, possess a relatively quick degradation time (approximately 4–12 weeks), which may result in a premature loss of structural support before the slow and complex cartilage healing process is organized. In contrast, PDO membranes exhibit a slower and more predictable degradation profile (approximately 6–12 months), providing prolonged mechanical stability and biochemical support throughout the extended phases of chondral repair and remodeling. This temporal compatibility between PDO degradation and cartilage turnover underscores its potential advantage over collagen membranes in maintaining extracellular matrix organization and functional regeneration in articular cartilage repair [7,8,29].

Adipose-derived MSCs (AdMSC) have emerged as a promising therapeutic strategy for articular cartilage lesions, combining abundant availability, multipotent differentiation potential, immunomodulatory activity, and the secretion of trophic factors that synergistically support tissue repair and regeneration [9,12,13]. The use of allogeneic AdMSCs has been investigated for a broad spectrum of clinical applications in regenerative medicine [16], offering key advantages such as standardized manufacturing protocols, rigorous quality control, ready-to-use (“off-the-shelf”) availability, sourcing from young and healthy donors, and inherently low immunogenicity. Collectively, these attributes not only facilitate large-scale production but also ensure consistent therapeutic quality, reproducibility, and broader clinical accessibility [16,34]. Oshima et al., [35] reported promising outcomes using a scaffold-free allogeneic AdMSC approach for treating osteochondral defects in the stifle trochlear groove of rabbits, demonstrating statistically superior results compared to the control group in macroscopic, histological, and immunohistochemical assessments for type II collagen. Although direct evidence was not provided, the authors suggested that the implanted allogeneic AdMSCs underwent differentiation into both, articular cartilage and subchondral bone following implantation into the osteochondral defect [35]. These findings are consistent with our results, in which the SadMSC/PDO group exhibited superior cartilage repair, primarily mediated by the abundant transplant of allogeneic AdMSCs, with no contribution from host-derived MSCs due to the absence of the MF technique.

The safety and efficacy of allogeneic AdMSCs for the treatment of osteoarthritis have been evaluated in phase 1 and phase 2 randomized clinical trials [16]. In a study performed by Sadri et al. [36] involving a single intra-articular injection of 100 million allogeneic AdMSCs in patients with knee osteoarthritis, significant symptomatic and functional improvements were observed over a 12-month follow-up period. These included notable reductions in the Western Ontario and McMaster Universities Osteoarthritis Index (WOMAC), Visual Analogue Scale (VAS), and Knee Injury and Osteoarthritis Outcome Score (KOOS). Additionally, structural benefits were reported, such as increased cartilage thickness in the medial anterior and posterior tibial regions. Importantly, the reported adverse events were mild and self-limiting, primarily localized swelling and pain at the injection site, without requiring specific medical intervention [36].

In this study, stem cell immunophenotyping was evaluated based on surface marker expression using RT-PCR, given the unavailability of specific antibodies for ovine cells in flow cytometry [19,20,37,38]. Despite Sanjurjo-Rodríguez et al. [39] recently describing flow cytometry for sheep bone marrow-derived MSC (SbmMSC) immunophenotypic characterization, they utilized antibodies anti-human (12 out of 15), anti-rat (2 out of 15), and only one (CD44) specific anti-sheep [39]. This action has the potential to compromise the validity and reliability of the assessment. Whereas McCarty et al. [40] demonstrated that commonly used human MSC surface markers (CD105, CD146, CD90, and STRO-1) do not exhibit cross-reactivity with SbmMSCs. Likewise, the absence of labeling for endothelial (CD31) and hematopoietic (CD45) markers further underscores this interspecies limitation. The absence of specificity significantly undermines the reliability of flow cytometry for ovine MSC immunophenotyping, highlighting the methodological constraints of directly extrapolating human antibodies to animal models [40]. In this context, RT-PCR emerges as a more consistent and informative approach for characterizing ovine adipose-derived MSCs (SadMSCs), enabling a more accurate assessment of their phenotypic profile.

The intrinsically limited regenerative capacity of articular cartilage, attributable to its avascular, aneural, and alymphatic nature, remains a significant challenge in the treatment of cartilage injuries. The microfracture technique is a widely utilized surgical approach for the treatment of chondral lesions due to its simplicity, cost-effectiveness, and minimally invasive nature. Microfracture accelerates scar tissue formation in the cartilage defect by inducing the formation of a clot over the injured area and mobilizing MSCs from the patient's own subchondral bone. The fibrocartilage formed lacks the biomechanical properties of native hyaline cartilage and tends to deteriorate over time, leading to symptom recurrence and limited functional improvement in the long run [[41], [42], [43], [44]]. Therefore, while microfracture remains a practical first-line option, its regenerative outcomes are suboptimal.

The microfracture technique allows bone marrow elements, including mesenchymal stem cells, growth factors, and other progenitor cells, to egress from the bone marrow cavity into the defect site. However, structural alterations in the subchondral bone can lead to long-term complications for patients. Adverse effects such as cyst formation, sclerosis, and bone overgrowth have been reported and are associated with impaired cartilage repair. These changes negatively affect both the quality and spatial distribution of newly formed cartilage within the defect, compromising the long-term success of regenerative strategies [[45], [46], [47], [48]]. For this reason, we advocate tissue-engineering approaches that do not rely on microfracture or deliberate exposure of the subchondral bone. A recent preclinical study using a human cartilage ECM-derived scaffold, with or without microfracture, in ovine full-thickness defects, demonstrated superior repair in scaffold-treated groups at both 6 and 12 months. Interestingly, the scaffold-only group showed reduced subchondral bone damage, underscoring the therapeutic potential of scaffold-based strategies over conventional microfracture [49].

In selected human patient populations, such as young individuals and athletes presenting with focal chondral lesions larger than 2 cm^2^, or failures in the prior treatment of articular cartilage lesions, the therapeutic objective extends beyond defect filling and aims at achieving high-quality cartilage repair that restores functional properties and long-term durability comparable to native hyaline cartilage [50,51]. Within this context, regenerative strategies with superior biological potential are indicated, particularly those combining a mesenchymal cell source with a 3D architecture entity, thereby providing a pro-regenerative microenvironment conducive to articular cartilage restoration [52]. The gold standard technique employed in these circumstances is the autologous chondrocyte implantation (ACI), which possesses 4 generations, adapting to patients’ needs and national regulations. However, this requires an initial arthroscopic procedure to harvest articular cartilage, followed by enzymatic chondrocyte isolation and expansion in a certified Good Manufacturing Practice (GMP) laboratory over several weeks, and a second arthroscopic procedure for implantation of the expanded cells. This two-stage surgical strategy increases patient morbidity, prolongs treatment timelines, and substantially elevates overall costs. Moreover, ACI demands a patient with no systemic disorders or chronic diseases, and faces additional regulatory challenges due to extensive laboratory manipulation and limited standardization of the final product [[50], [51], [52], [53]].

In comparison, the combination of a PDO membrane with allogeneic AD-MSCs offers off-the-shelf availability and outstanding manufacturing versatility, reducing cost and preparation time, and greater adaptability to regulatory requirements for cell-based therapies due to AD-MSCs allow for stringent quality control and product standardization, with continuous testing to ensure safety and efficacy prior to clinical application [7,12,26,28]. Thereby represents a more practical and scalable solution with a strong promise for the effective restoration of chondral and osteochondral lesions. Although safety and efficacy have been demonstrated in preclinical studies and randomized clinical trials [16,35,54], the clinical translation of this therapeutic approach remains primarily constrained by regulatory frameworks, particularly those restricting the use of ex vivo–manipulated, allogenic, and laboratory-processed cell-based products.

ICRS macroscopic scoring systems offer a rapid, gross overview of surface integrity and defect filling of the cartilage repair; however, they lack the resolution needed to assess the structural organization, cellularity, and matrix composition of the newly formed tissue [55]. In our study, the COL/MF and PDO/MF groups achieved superior macroscopic scores compared to the isolated MF group and non-microfracture groups (Control, SadMSC/COL, and SadMSC/PDO). The rapid filling of the cartilage defect induced by the microfracture technique was further augmented by integration with the PDO scaffold. This effect is likely attributable to the scaffold's structural support, which enhances the distribution and stabilization of the forming clot, promotes the retention of MSC and growth factors at the defect site, minimizes cellular and mechanical loss of the clot into the joint cavity, protects the subchondral bone, and provides a physical and biochemical microenvironment that is more conducive to the migration, adhesion, and chondrogenic differentiation of host bone marrow-derived cells [52,56]. The findings of this study align with those reported by Migliorini et al. [57], who compared different surgical procedures for the treatment of focal cartilage lesions in the human knee. In their analysis, the combination of microfracture and a scaffold demonstrated the most favorable short-term clinical performance, as evidenced by outcomes assessed over a 3-year follow-up period. Notably, the PDO membrane demonstrated significantly better macroscopic outcomes than microfracture alone, supporting its potential to optimize cartilage repair.

Histopathology remains the most specific and informative technique, providing in-depth analysis of cellular and extracellular matrix organization. It allows the identification of features such as hyaline-like cartilage formation, integration with native tissue, tidemark presence, and subchondral bone changes. Histologically, the highest repair quality score was observed in the SadMSC/PDO group, exhibiting superior proteoglycan content, as evidenced by Safranin O/Fast Green staining, followed by the PDO/MF and MF groups. Although the SadMSC/PDO group showed the highest median histological score, it did not reach statistical significance due to large within-group variability. This suggests that the quality of repair may vary among individuals, and future studies with larger sample sizes are needed to confirm this trend. Similar Modified O'Driscoll scores have been reported by Beck et al. [48]. The discrepancy between macroscopic and microscopic outcomes likely reflects the slower progression of hyaline-like tissue formation in the MSC scaffold-seeded MF-free strategies, resulting in a lower ICRS macroscopic score, along with the study limitation of the single time-point assessment at 26 weeks, which may have been insufficient to capture full repair tissue maturation. Clinical studies and systematic reviews indicate that different cartilage repair strategies yield distinct patterns of tissue maturation and histological quality over time. Scaffold-associated and cell-based techniques have been shown to support the development of more organized, hyaline-like repair tissue compared with the predominantly fibrocartilaginous tissue produced by MF, in which the clinical differences become pronounced at medium to long-term follow-up [52,58]. Histopathological evaluation supports PDO scaffolds as safe biomaterials that improve repair quality, especially when combined with SadMSCs.

Immunohistochemistry has become an essential tool for evaluating cartilage repair, offering insights into molecular composition directly linked to repair durability [22,23,49,59]. Alcaide-Ruggiero et al. [23] proposed a semi-quantitative scoring system for sheep, assessing staining pattern and intensity on a 1–3 scale. COL I is absent in healthy cartilage but expressed in fibrocartilage and pathological conditions [23,59]. In our study, the MF group showed predominant COL I labeling, indicating inferior fibrocartilaginous repair. COL II, the principal ECM protein of healthy cartilage (90–95%), was most strongly expressed in the SadMSC/PDO group, supporting the hypothesis that cell-seeded PDO scaffolds generate tissue more closely resembling native cartilage [23,49,59].

Type X collagen is widely known as a marker of hypertrophic chondrocytes and dystrophic mineralization in articular cartilage [59,60]. In this study, COL X staining was restricted to the deep cartilage layers across all groups, consistent with its role in reestablishing the cartilage–bone interface at 26 weeks. Chondrogenic differentiation of MSCs is largely mediated by the TGF-β family, key regulators of ECM synthesis [61]. TGF-β2 initiates chondrogenesis, whereas TGF-β3 supports stable hyaline-like cartilage repair [[62], [63], [64], [65]]. The MF group exhibited the lowest TGF-β2 and TGF-β3 expression, suggesting limited chondrogenic potential. Conversely, SadMSC/PDO showed markedly higher expression of both isoforms, underscoring its superior capacity to promote chondrogenesis and cartilage regeneration. Molecular profiling confirmed that SadMSC/PDO-treated cartilage more closely resembled healthy cartilage, not only structurally but also biochemically.

Based on the findings from Picrosirius red staining and the analysis of collagen fiber orientation within the repaired cartilage, the SadMSC/PDO-treated group demonstrated the most favorable regenerative profile, exhibiting structural features more closely resembling those of native cartilage tissue. Specifically, the parallel alignment of collagen fibers in the superficial zone relative to the articular surface is associated with enhanced shear resistance, while the perpendicular orientation observed in the deeper layers contributes to improved load distribution and shock absorption during locomotion [3,25]. Although no statistically significant differences were observed in the macroscopic evaluation among groups, the integration of histological findings with collagen fiber organization suggests superior functional and structural properties in the SadMSC/PDO-treated group.

The quality of cartilage repair is a critical determinant of long-term outcomes. Puhakka et al. [66] reported a moderate correlation between arthroscopic and histological ICRS scores in porcine models, highlighting the limitations of gross evaluation alone. In our study, the multimodal evaluation, combining ICRS macroscopic scoring, Modified O'Driscoll histology, and immunohistochemistry, provided a comprehensive assessment of the articular cartilage repair. We found a weak correlation between ICRS and histology, suggesting that macroscopic scores alone are insufficient predictors of repair quality. Strong positive correlations of COL II and TGF-β isoforms with histology indicate their utility as molecular markers of high-quality repair, while COL I deposition inversely correlated with advanced chondrogenesis markers (COL X, TGF-β3).

While this study presents important insights in the domain of cartilage repair, certain limitations should be considered. The single time-point assessment of cartilage repair at 26 weeks and the absence of an evaluation of the inflammatory profile of synovial fluid may have restricted a more comprehensive understanding of the biodegradation dynamics, biocompatibility of the evaluated membranes, and made it not feasible to directly evaluate the inflammatory or anti-inflammatory effects of the treatments. In addition, the absence of a negative control group for the immunohistochemical analysis limited the ability to establish baseline marker expression. The inclusion of an appropriate negative control group in future studies would strengthen the interpretation and reliability of the immunohistochemical findings.

In conclusion, the findings of this study indicate that the PDO scaffold exhibits excellent articular biocompatibility and biodegradability when applied in articular cartilage tissue engineering, and when combined with allogeneic AdMSCs, significantly enhances the quality of cartilage repair in a sheep model. While PDO/MF achieved the best macroscopic outcomes, SadMSC/PDO provided the most favorable microscopic and molecular findings, suggesting that allogeneic AdMSCs seeded on PDO scaffolds enhance hyaline-like cartilage formation. Compared to the COL scaffold, PDO provided more steady and durable structural support due to its slower and predictable degradation profile. PDO-based constructs may represent a promising alternative to conventional MF and possess a strong translational potential for clinical application in cartilage repair strategies.

## Ethics statement

This study was approved by the Ethics Committee for the Use of Animals in Research (CEUA) of the School of Veterinary Medicine and Animal Science of the São Paulo State University – UNESP (0068/2021-CEUA).

## Data availability

The raw data underlying the findings of this research will be made available by the authors upon request. Data access inquiries should be directed to Dr. Apolonio (e.apolonio@unesp.br) or Dr. Alves (ana.liz@unesp.br).

## Declaration of generative AI in scientific writing

No generative artificial intelligence (AI) or AI-assisted technologies were used in the preparation of this manuscript.

## Declaration of competing interests

The authors declare that they have no competing financial interests or personal relationships that could have influenced or could reasonably be perceived to have influenced the work reported in this manuscript.
