# Supplementary material for: Combining polydioxanone-based scaffold and allogeneic adipose-derived mesenchymal stem cells enhances articular cartilage repair - a pre-clinical study
Source: J Orthop Translat. 2026 Aug 25;60:101193. doi: 10.1016/j.jot.2026.101193 (PMC13527560; doi:10.1016/j.jot.2026.101193)
Supplement: Supplementary file 1 [file mmc1.pdf]

Supplementary Materials

Figure S1

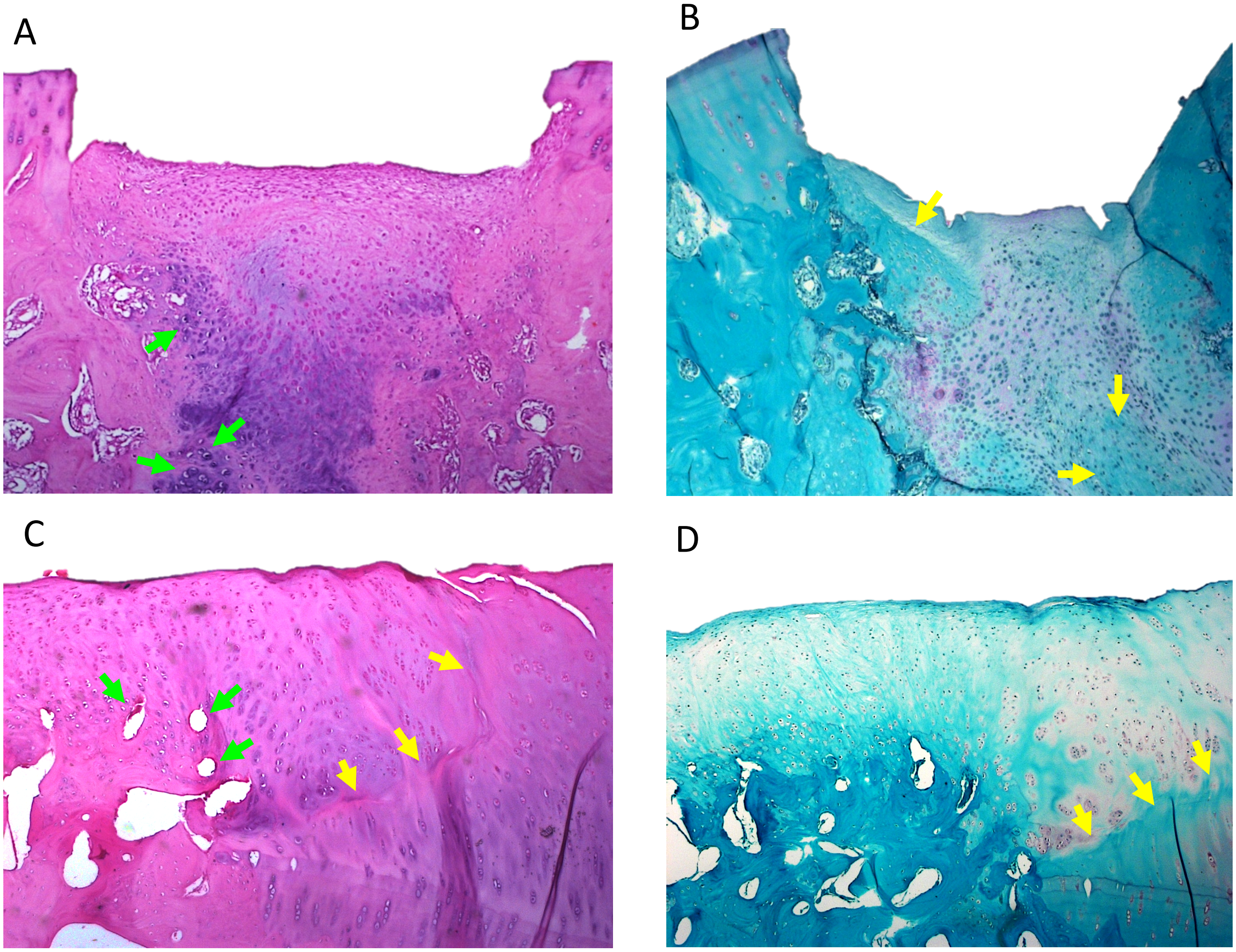

Figure S1. Histopathological findings of cartilage repair across experimental groups. **A.** The image corresponds to the MF group and illustrates hypertrophic chondrocytes (green arrows). **B.** Represents the MF / COL group and illustrates the fibrocartilage deposition stained with Fast Green (yellow arrow) along the microfracture tract. **C and D**. Images correspond to the MF / COL group and highlight the presence of blood vessels in the repaired cartilage deep layer (green arrows) and demonstrate the interface between healthy cartilage and fibrocartilage (yellow arrows) within the repair tissue of the chondral defect.
